# Spirit of the English Periodicals, and Notices of English Medical Literature

**Published:** 1836-04-01

**Authors:** 


### Periscope;

OR,

### CIRCUMSPECTIVE REVIEW.

" Ore trahit quodcunque potest, atque addit acervo."

### I.

### Spirit of the English Periodicals, and Notices of English Medical Literature.

### On Diseases of the Spleen. By Mr. Twining.

This disease is very frequent in Bengal, and in consequence of our frequent communication with the East, and the great influx of Anglo-Asiatics into this country, splenic enlargements are not very rare occurrences even on our own soil. We propose, therefore, to give here, a very brief and condensed abstract of Mr. Twining's experience on this subject.

Vascular engorgement generally precedes induration of the spleen, and both states present symptoms of an inflammatory nature. Mr. T. has never seen suppuration of that organ. There is usually tenderness on pressure, in the region of the spleen. Acute inflammation of the peritoneal coat of the spleen is a rare disease in any country. Mr. T. seems to regard disease of the spleen not so much an idiopathic affection, " as one of the attendant phenomena on a peculiar constitutional disorder." The characteristic features of this disorder are general debility, pallor, deficiency of red blood in the capillaries—hectic blueness or pearl colour of the sclerotica —chlorosis of the visage, tongue, and gums. At a more advanced period, the circulation is languid, and the extremities cold—skin pale, shrivelled, and arid, or furfuraceous.

Tumid spleens are often borne with little apparent inconvenience, the individuals being able to walk about, and eat like other people. But the disease is most formidable in children, ending too often in general decay of health and progressive marasmus. Their breath has a peculiarly disagreeable odour. The disease is by no means confined to the natives. It frequently affects the Europeans, especially after fevers. Patients of this kind are very liable to sloughing ulcers from slight bruises or wounds. Blood drawn from splenic patients sometimes coagulates imperfectly, and presents no serum—sometimes the cruor is black and soft—seldom exhibits a buffy coat, except when pyrexia is present. The serum, when healed, coagulates as firmly as that of a healthy person. Many symptoms of scurvy are present in cases of vascular engorgement of the spleen;—such as tendency to hæmorrhage from various external and internal parts, &c. Most patients also labour under difficult respiration—impaired appetite—slow digestion—imperfect assimilation—despondency—inactivity, &c. In the absence of active pyrexia, the urine is pale, and often copious. In the latter stages there is œdema of the feet, and sometimes of the face. In the majority of fatal cases, the patient dies of dysentery or ascites.

Splenic tumefaction sometimes takes place very suddenly in the course of remittent and intermittent fevers in Bengal, and in other low and marshy localities. Its extent is very various—sometimes reaching to the navel, or even filling the whole abdomen.

The post-mortem appearances are thus classified by our author.

" 1.—A soft rounded enlargement of the spleen, the texture less firm than in the healthy state, and easily broken if the finger be pushed abruptly against it. In some cases the part is so much softened, that it resembles a great clot of blood wrapped in a thin membrane: this varies in color, from black to brown or blue; and in the extreme degree of softening, when we attempt to lift the tumid spleen, the fingers are thrust through the membrane, and the organ breaks down in the hands, becoming a putrid gore. This soft globular enlargement from vascular engorgement of the spleen, most commonly attends, or follows, the severe remittent fever of the rains and cold season, when that disease attacks weak and unhealthy young persons.

2.—Oblong enlargement of the spleen; the organ being more firm in texture than in its natural state, its edge thin and notched; the color being sometimes a pale brown, though more generally a dusky red. This morbid change of structure would appear to be the result of more slow and gradual degeneration, which in its earlier stages has probably been attended with some inflammatory condition of the internal structure of the spleen; in such cases we also find evidence of superficial inflammation, attended with adhesions to adjacent parts, more frequently than in the rounded enlargement from simple vascular engorgement.

3.—Opaque patches of various sizes: some of these extend over half the convex surface of the spleen, and are nearly ⅛th of an inch thick; they may be deemed the result of albuminous depositions during superficial inflammation.

4.—Adhesions of the peritoneal coat of the spleen to contiguous viscera; which adhesions are by no means a general result of tumid spleen in Bengal.

5.—In a few old cases, we find a more indurated friable spleen, that breaks when handled without much force, like a piece of old moist cheese.

6.—Still more rare is the firmer induration intersected with septa of condensed fibrous structure, to which we give the name of scirrhus.

7.—Tubercles of various sizes, generally small, and of grey or brown color.

8.—An organised coagulum in the splenic vein.

9.—Encysted tumors.

10.—Abscess of the spleen is said occasionally to occur: but I have not yet seen an example of suppuration in the spleen, in Bengal."

In respect to vascular engorgement of the spleen, purgatives combined with bitters and steel, have proved most useful. The following is Mr. Twining's usual formula:—

℞. Pulv. jalap.

—— rhei,

—— calumbæ,

—— zingib.

Potassæ supertart. āā ʒj.

Ferri sulph. ℈ss.

Aquæ menth. sat. ℥x.

Misce.

An ounce and a half, (for an adult,) twice a day—at 6 in the morning and at 11 forenoon. It is said to act as a purgative, tonic, and diuretic.

The natives have a decided conviction that tonics are useful in enlargements of the spleen, and that the astringent effects of iron are produced in the splenic vessels by a kind of imbibition from the stomach.

In chronic enlargements and induration of the same organ, similar remedies appear to answer, but they are more uncertain in their effects. The following is a curious circumstance, if a fact. "If the tumid spleen be of a *globular* shape, an enormous degree of disease may generally be cured by the remedies already pointed out; but if the enlarged and indurated spleen be of an *oblong* shape, with a thin sharp edge, deeply indented with notches, a cure is much more difficult, and cannot generally be expected, except as the result of a careful and prolonged treatment, sometimes requiring the aid of a sea-voyage."

The daily application of leeches, at the beginning, is beneficial, when combined with the purgatives and tonics above-mentioned. To these must succeed blisters or issues. It is curious that hepatitis is very rare among the natives, though so common among Europeans in Bengal; while spleen-disease is extremely common among the *former*, and comparatively infrequent among the *latter*. The remedies are the same for both. The natives use acupuncture and the actual cautery—and as their remedies are the result of observation and not of reasoning, they are not to be entirely despised. Mercury seems to have a most injurious effect in diseases of the spleen, producing extreme debility, depression, and exhaustion, with destructive salivation, ulceration, and sloughing of the gums, lips, &c. This circumstance renders very difficult the treatment of complications of hepatic and splenic enlargements. Mr. Twining passes a severe censure on Mr. Annesley for advocating the use of mercury in diseases of the spleen. We have no doubt that the spleen diseases of Bengal, like many other diseases of that part of India, differ considerably from the same diseases in the Peninsula, and especially in the Madras presidency. This may account for the discrepancy of practice between two experienced practitioners.

---

Cessation of Pulsation in several large Arteries of the Upper and Lower Extremities. By Mr. Ed. Crisp.

This is one of the most remarkable cases, of the kind, on record. We have had an opportunity of carefully examining the patient, through the kindness of Mr. Crisp, and shall now lay the particulars before our readers, omitting the minute details of daily prescription.

A young female, aged 22, of healthy parents, had had a severe attack of pneumonia about three years ago, under Mr. Crisp's care. Subsequently she was in service at Hackney, where she had been exposed to cold, and never considered herself as quite well after the pneumonia. She had head-aches, and occasional fits of hysteria.

*Jan. 31st*, 1835. She came under the reporter's care, complaining of pain in her legs and arms, with rigors followed by febrile action. Pulse small and 90 in number—bowels confined—tongue furred—pain in the epigastrium—menses regular. Aperients and febrifuges. These lessened the epigastric pains, but those of the lower extremities continued. On the 3d of February, Mr. Crisp was sent for, on account of severe pain in the stomach, with vomiting. The pain had left the lower extremities. She was bled to eight ounces, with relief. 4th. The pains returned in the extremities, and ceased in the stomach. 5th. Hands and arms cold, with tingling sensation in the fingers. *No pulse at either wrist*—heart and carotids acting strongly. On the 9th she was examined. Pulsation ceased about an inch below each clavicle—dorsal arteries of the feet beating forcibly at 90. 11th. The integuments of the right arm extremely painful. Dr. Whiting visited her, and found pain and tenderness, on pressure, along the main arteries of the arms. Ordered leeches below the right clavicle, and to have calomel and antimony.

13th. *No pulsation* could be felt in the dorsal arteries of the left foot, and indistinct in the popliteal. A thrilling sensation in the carotids, and *bruit de soufflet* over the heart. 14th. A slight vibratory movement felt in the radial artery of the left arm. 16th. Pain of left leg severe. 18th. No pulsation in either of the arms—ceases about three inches below Poupart's ligament in the left thigh. The foot and fore-part of the leg of a purple colour—tips of toes cold—profuse perspiration over every part of the body. 22d. *No pulsation in any artery of the extremities.* The toes and sole of the left foot black. March 6th. Since last report the pain has become excessive—very little sleep—pain in the region of the heart—bruit de soufflet—little and great toe of *right* foot black and painful. These were removed on the 8th March. We need not pursue the details of this curious case. On the 5th of October the left leg was removed below the knee. On slacking the tourniquet, there was very little bleeding from the large arteries—"*the blood not coming per saltum.*" The smaller arteries bled freely, and it was necessary to take up nine or ten of them. The leg was examined with great care. The large arteries were slit up, "but no trace of disease could be found in them." "They appeared to be smaller than natural."

We examined this young woman on the 21st December, in company with Mr. Crisp and Mr. Hooper, and noted the following particulars.

The stump is nearly healed. The temperature of all the extremities is considerably below par. The left arm has the highest temperature. In it there is a very faint pulsation of the radial artery. In none of the other extremities could we detect any action of arteries by the finger or the stethoscope. The subclavian of the *right* side presented the loudest *bruit de soufflet* we ever remember to have heard. The same in the right carotid. In the right axilla no pulsation could be felt or heard. The *left* subclavian gave out considerable *bruit de soufflet,* but less loud than on the opposite side. In the left axilla, a faint pulsation could be heard by the stethoscope. The *bruit* in the *left* carotid was in proportion to that in the subclavian of that side. The action and sounds of the heart were perfectly normal. The muscular strength of the limbs corresponded with the temperature and state of the circulation. It was very weak in all the limbs, but strongest in the left arm, where some pulse was still perceptible.

The case excited considerable discussion in the London Medical Society—some of the members contending that the loss of circulation, or at least of pulsation, was a functional disorder, of an hysterical nature. Others maintained that there was a mechanical obstruction to the circulation, occasioned by arteritis or some other cause. Among the latter we ranged ourselves, and we have not the smallest doubt that a mechanical obstruction of the circulation exists.

In consequence of the interest excited by this case, we have tried back for illustrations. The following is the result of our researches, though doubtless a more diligent inquiry would disclose many other examples of an analogous kind.

Dr. Parry, in his work on the pulse, page 140—says that he has met with twenty instances of cessation of the pulse—some terminating fatally, others favourably, after a longer or shorter period. Only one dissection, however, is given.

"A female, affected with severe cough, was apparently convalescent, when it was discovered that the pulse in one arm was wholly wanting. A few days afterwards she died suddenly.—*The whole course of the aorta* was carefully examined; but no deviation from the healthy state could be perceived in it."

In the 19th vol. of the Med.-Chir. Rev. p. 285, a curious case is communicated by Dr. Ferguson, of the 5th Regiment of Foot. A young, but delicate soldier, was sent on escort duty in Ireland, where he got thoroughly drenched with rain. On his return, he was seized with violent pneumonia, accompanied by distressing palpitation of the heart, and a *total absence of pulse in both wrists.* The action of the carotids, in the mean time, was unusually strong. The inflammation of the lungs was subdued, but the abolition of the pulse in both the upper extremities continued many months afterwards, when the case was communicated to Dr. Johnson. During this time he was unable to do any duty, and could take very little nourishment, yet he did not lose flesh. He was subject to severe pains under the sternum, with sense of suffocation, and livor of the countenance.

Morgagni quotes a case from Ramazini, of a young man in whom abolition of pulsation in all the arteries existed for four days. During these four days he was cold and passed no water. He died, but no dissection is given.

In the Medico-chirurgical Review for December, 1822, Dr. Johnson has given an account of a case of non-pulsation in all tangible arteries, except the carotids, where a very faint pulse was felt. On examining the heart, no action could be felt or heard in that organ! The patient laboured under hydrothorax, ascites, and anasarca. The pulse returned on the third day, after brisk purgation, and considerable diuresis. He died, and presented dropsy of chest and abdomen, with organic disease of the heart. The pressure of fluid was supposed to embarrass the action of the heart, and prevent the evidence of pulsation there, or in the arteries for a time.

The first thing that attracted John Hunter's attention to his own case, was temporary loss of pulsation in the wrists. In one of these states, he happened to look in a glass, and perceived that his face was like a corpse. He died in one of these attacks, and it was found that he laboured under angina pectoris. The heart was small and flabby—the coronary arteries and mitral valves ossified.

Sir B. Brodie mentions a remarkable case in his lectures. "A man was walking on a hot day through Battersea-fields, when he experienced pain in the lower extremities, one of which was more affected than the other. The pulse disappeared in this extremity—the foot mortified—became dry, hard, and horny. The mortification reached to the middle of the thigh, and there stopped; but the man died in St. George's Hospital. On dissection, both artery and vein were found inflamed, from the middle of the iliacs to the middle of the thigh, and their canals filled with coagulable lymph."—*MS. Lect.*

Sir B. B. quotes a case of this kind from Saviard, who amputated, but had not a single artery to tie, all being choaked up with coagulable lymph.

In the 4th vol. of the Med.-Chir. Rev. there is related a remarkable case of non-pulsation in all tangible arteries during a period of 24 hours. The gentleman (Mr. Lowndes) is now residing in the Haymarket. From excessive urethral irritation, a most serious constitutional disturbance was set up. The pulse disappeared from all tangible arteries—the extremities were cold—the fingers blue—breathing short and laborious. On careful examination with the stethoscope, in company with Mr. Thomas of Leicester-place, only an indistinct fluttering of the heart could be perceived—and no action of any of the arteries. The patient was, in fact, in the same condition, as respects the circulation, in which so many thousands of people have been found during the late epidemic cholera. The heart was clearly in fault in this case—and stimulants restored the circulation at the end of 24 hours.

In the 19th volume of the Med.-Chir. Review, p. 207, the following case is related from the *Archives Générales.*

A girl, 17 years of age, became affected with pain in the right lower extremity, and she entered La Charité 14 days after the commencement of the complaint. On examination, no pulsation could be perceived in anterior or posterior tibial artery, nor in the popliteal artery. The disease ultimately ended in dry gangrene and death. On dissection, the crural artery, from the groin to the ham, was converted into a hard cord, plugged up by a coagulum. There were marks of arteritis in various parts of the arteries of this extremity.

In respect to Dr. Parry's case, it is hardly necessary to remark, that the examination of the *aorta* led to no satisfactory results. It was like exploring Fleet-street, to find out the cause of an obstruction in Holborn!

---

### On the Functions of the Fœtal Kidney. By Robert Lee, M.D.

There is no positive information, Dr. Lee observes, in our systematic works on physiology, respecting the function of the kidney previous to birth. All the glands employed in the digestive process have considerable volume in the fœtus, and may be supposed to possess some activity. Abernethy did not think that the fœtal kidney secreted urine. The following fact is brought forward by our author, as decisive of the question.

"On the 2d of January, 1835, Mr. Hay, of Osnaburgh Street, attended a patient who was delivered in the 8th month of a still-born female child. It had a double hare-lip, both its feet were clubbed, and the abdomen was so large that it passed with difficulty through the pelvis. Mr. Hay examined the body on the following day, and he found the distention of the abdomen to arise from an accumulation of fluid within the kidneys, produced by an impervious state of the ureters. The right kidney, which resembled a thin cyst filled with a watery fluid, was larger than the head of the child, the left did not exceed half this bulk. Both kidneys were removed from the body without the fluid they contained having escaped, and were in that state presented to me by Mr. Hay. An opening having been made into the pelvis of the left kidney, ℥iv. of a fluid resembling urine flowed out; the pelvis of the right kidney contained nine ozs. of a fluid having the same appearance, which was examined by Dr. Prout.

The following letter from Dr. Prout contains an account of the chemical composition of this fluid.

'DEAR SIR,

I send a short account of the fluid from the kidneys of a fœtus in whom the ureter was found impervious.

The fluid was of a deep brown colour, somewhat like diluted porter or table beer, transparent, and without any remarkable smell. Specific gravity about 10·12. Very slightly acid. On exposure to heat, it became opaque and deposited flakes of albuminous matter, which was next examined by the addition of an acid. The deposit was of a deep brown colour. The separated fluid was nearly colourless, and deposited on cooling a considerable quantity of lithic acid crystals.

When evaporated to dryness and treated with alcohol, that menstruum was found to take up a principle strongly acid, and which assumed readily an imperfect crystallized form. I cannot venture to give this principle a name; it somewhat resembled the acid called amniotic, or rather allantoid, in some of its properties, but differed from it in others. The alcoholic solution gave at first faint and somewhat doubtful traces of urea; on standing several days these became very distinct. After the albuminous matter was separated, ammonia produced a deposition of triple phosphate.

These results prove beyond a doubt that the fluid was of an urinary nature, and render it probable, that as the liver in the fœtus secretes bile, so the kidneys secrete urine long anterior to birth; and that in a perfect state of the organs the fluid is constantly escaping through the bladder and mixing with the amniotic. This fact has been often suspected, or rather taken for granted, but has never to my knowledge been proved.

I send a portion of the fluid containing a deposition of lithic acid crystals.

Yours,

*Sackville Street*, W. PROUT.'
*Jan.* 9, 1835.

On the 12th February, 1835, Dr. Lee induced premature labour for a deformed pelvis, in a case of six months' pregnancy. Thirty-two ounces of pure liq. amnii flowed through a silver catheter. It was of a straw colour, neither acid nor alkaline. Neither Dr. Bostock nor Dr. Prout could discover any urea or uric acid in it. This renders it probable that, at six months' utero-gestation, a very small quantity, if any, urine is formed by the fœtal kidney.

Mr. Howship has pointed out some curious cases from his treatise on the urinary organs, where urine was found in the bladders of children born before the full term. Sir B. Brodie also communicates a case which seems to decide the point. A male fœtus was brought into the dissecting-room, of nearly the full time. The external orifice of the urethra was impervious, and the bladder was found to be moderately distended with urine, as were the infundibula and pelvis of each side. The urine was examined by Mr. Brande, who proved that it contained the usual properties of urine, except uric acid. Other instances are also quoted. The inquiry is curious in a physiological point of view.

---

THE RUSSIAN PUZZLE.—"PERICARDIAL EXUDATION."

Under the latter title, Dr. Seidlitz, of St. Petersburg, has described a disease which, we are confident, no one has either seen or described in this country. Our indefatigable contemporary (the BRITISH and FOREIGN) has ferreted out, with transcendental olfactories, this precious morçeau of pathological novelty, and from his pages we shall quote one of the cases as a specimen of the whole.

" Towards the end of February (1831), a sailor, aged 27, of a muscular habit, was seized with an unusual degree of lassitude after a hard day's work. A peculiar sense of anxiety caused him repeatedly to start out of an unrefreshing slumber. Tightness of the chest ensued on the following day. The præcordia seemed the primary seat of distress, from whence an obtuse pain radiated over the whole anterior surface of the thorax, and concentred itself on the second day (that of his admittance at the hospital) in the right hypochondriac region. Both these places were impatient of touch, which, without precisely causing pain, seemed to the patient to impede his breathing. The countenance was bloated, of a bilious hue. The breathing was regular, but superficial; deep and full when the patient exerted himself, and attended by neither pain nor cough. Pulse very frequent, and very indistinct (suppressed, unterdrückt). Pulsation of the heart imperceptible to the hand, and scarcely audible. The patient was perfectly conscious, but morose and torpid, preferring to lie flat on his back or on his left side, and with his head low. He denied having been attacked with rigors or with febrile heat. VS. ad ℔. jss. Blood buffed, yielding a large proportion of serum. A solution of nitrate of potash with tartarized antimony, and saline clysmata, were administered. The latter produced several stools. The night was passed more tranquilly, although but with little sleep. The pulse had now become less frequent and more developed, but the pulsation of the heart was more indistinct. The countenance was expressive of great anxiety, but still nothing was complained of but an entire prostration of strength. The temperature of the skin underwent frequent changes from moderate warmth to a marble-like coldness, accompanied by a cold sweat. Action of the kidneys regular. Four cupping-glasses were applied to the region of the liver, and the saline potion and clysmata repeated. On the morning of the fourth day, the patient's apathy had increased: alternately groaning and sighing, he evinced a great dislike to be interrogated. Pulsation, both of the heart and arteries, was no longer distinguishable; the lips were blue; face and extremities of a marble-like coldness. Whilst Dr. S. was in the act of examining the abdomen, the patient suddenly applied his left hand to his head, then to his chest, stretched his eyelids wide asunder, and expired. In the next moment the pupils became strongly dilated. An incision was instantly made into the swollen jugular veins, from which the blood issued in a small stream. Respiration was at the same time artificially sustained by alternately compressing the thorax, and again allowing it to expand, but in vain.

On the thorax being opened, the eye was struck with the extraordinary size of the pericardium, and with its livid appearance, owing to the translucent fluid wherewith it was distended. An incision being made into this membrane, from four to five pounds of fluid escaped, having the colour of dark venous blood and the consistency of a decoction of marsh-mallows. The entire absence of crassamentum disproved this fluid to be genuine blood, whilst the fact of its coagulating through the influence of heat, showed it to consist of the albuminous serum mixed with the colouring matter of the blood. The inner surface of the pericardium was tinged of a dark blue red, and lined with a coat of villous, granular fibres, of the same colour, which lining was easily separated from the pericardium, and had rather the character of a deposit from the fluid than of the false membranes which attach themselves to the pleura in exudatory inflammation of the latter membrane. On removing the above fibrous layer, the envelope of the heart appeared of the same blue red colour; its surface smooth, shining, and sound. The substance of the heart itself was dark red and very firm. The compression by the extravasated fluid had reduced the volume of the heart to the size of a man's fist, and its four cavities to a mere nothing. The abdominal organs were healthy, although the veins were here, as well as in the thoracic cavity, gorged with blood."

Another case is related, where, on opening the thoracic and abdominal cavities, several pints of blood flowed away—and in the pericardium itself were four pints of bloody fluid, without crassamentum. There was a coat of fibrine on the peritoneal surfaces, but the membrane itself was sound.

At a later period (1832) some cases were successfully treated by depletion and derivatives.

Our readers, we think, will agree with us, that the above disease is a puzzle. It more nearly resembles the BERRIBERRI of India, than any other disease with which we are acquainted. It is a peculiar cachexy, which we are not likely to witness in this climate, and must be owing to causes of which we are entirely ignorant.

---

### TEMPERATURE OF DIFFERENT PARTS OF BODIES IN A STATE OF DISEASE.

Messrs. Bequerel and Breschet have made a number of experiments on the foregoing subjects—though we confess that we can see little other object in such experiments, except the gratification of curiosity. And, considering the numerous grave subjects in physiology, pathology, and therapeutics, which yet remain open to investigation, we are rather surprized that the more important inquiries should not take precedence of the less important. Mais, chaque à son gout. We shall state one or two experiments. A man had typhoid fever, complicated with bronchitis. The temperature of the mouth was 102°—while that of the biceps muscle of the arm was about a degree and a third higher. Again. A woman had a cancer of the breast. The temperature of the mouth was 98°—ditto the cancer—ditto the biceps muscle.

Now, we do not deny that these may be very important physiological facts. All we can say is, that we are too dull to recognize their importance. Perhaps some of our readers may be more fortunate.

### ANOMALOUS CASE OF LITHOTOMY. By PROFESSOR LIZARS.

A surgeon attached to a large hospital has peculiar opportunities for improvement, and he fails of his duty, if he either neglect them, or conceal the useful results of his observation and experience. Success, though it flatters vanity, is at best an equivocal proof of merit, for it may happen to the rash and unskilful. Successful cases, in ordinary circumstances, when published, afford but little information; timidity may be unwilling to divulge the unsuccessful, but these are for the most part our proper instructors; from these we learn whether nature or art is the more to be blamed for any untoward event; but whether successful or unsuccessful, those cases are invaluable which lead to the detection of such morbid deviations as would certainly occasion the death of a patient in the hands of an irresolute operator. Of this last class is the following case.

Jas. Brown, a healthy-looking man, fifty-nine years of age, entered the hospital on the 29th December last, and presented the usual symptoms of stone in the urinary bladder, under which he had laboured during the last eight months. He had been in this hospital seven months ago under the late Professor Turner, who sounded him, but detected no calculus. The day after his admission he was carefully sounded, but no stone was felt; the bladder was rough and fasciculated. He was ordered warm baths, leeches to the region of the pubes, the mistura aquæ potassæ, the uva ursi, and a seton over the pubes. By these means all irritation having been subdued, he was again sounded, and a stone distinctly perceived. A dose of castor-oil was administered; and on the following day the lateral operation was performed.

All the preliminary steps having been taken, and the existence of a calculus again ascertained, a large staff was inserted, which could not be made to pass the prostatic portion of the canal. A smaller staff was next employed, which apparently entered the bladder, as its handle was loose and moveable. One of my colleagues held it over the pubes, whilst I commenced, and cut down to the membranous portion of the urethra. I then proceeded seemingly through the left lobe of the prostate gland, which was hard, cartilaginous, and studded with calcareous deposition. The left fore-finger, which guided the lithotomy knife, seemed to enter the urinary bladder, and a little fluid, considered to be urine, flowed out, when I begged the staff to be withdrawn. I next inserted a pair of forceps; but instead of a calculus such as the sounding had led me to expect, I discovered nothing but calculi, varying in size from that of a millet-seed to that of a pea. I now used a searcher, but was not more fortunate. My finger felt a pouch equal in magnitude to a urinary bladder, which contained numerous small calculi. One of my colleagues, at my request, introduced his finger, and the sensation communicated so nearly resembled that of a mucous membrane, that he suspected I had wounded the rectum, but convinced himself of the contrary by examining that viscus with the forefinger of his other hand. Another of my colleagues was also requested to examine, and he, with a scoop, removed some of the small calculi already mentioned.

I now inserted a catheter, which passed the entrance of this pouch, got into the bladder, and urine flowed out. The catheter was replaced by a staff, along which the knife was carried through the neck of the bladder, as there was no substance like prostate gland, and a stone of the size of a flattened plum was instantly extracted.

The first incisions into the pouch occupied about one minute. The second incision and extraction of the calculus about another minute. From fifteen to twenty minutes were spent in examining this pouch.

The patient has had no bad symptom —no case of lithotomy ever went on more favourably—and this is now the tenth day from the operation.

The anomalous pouch, which rendered this case so complex, seems to me to have been nothing more than the external fibrous capsule of the left lobe of the prostate gland gradually dilated, until it became as large as the bladder itself.

Crosse, in his work on Urinary Calculus, p. 34, says, " Concretions in the prostate gland, commencing in its ducts, often at a distance from their urethral orifice, even at the very bottom of a duct, go on increasing until each duct is enlarged into a pouch, rendering an escape of the concretion into the urethra impossible; the narrow orifice by which the pouch communicates with the urethra becomes often closed, in consequence of inflammation and effusion of lymph; the pouch is a secreting cavity which furnishes additional deposit; and as the concretions enlarge or multiply, the pouch enlarges in the direction of where there is least resistance towards the lateral or posterior surface of the prostate gland."

Wilson, on the Urinary Organs, at page 353, also observes, " I have met with a urinary calculus larger than a common-sized olive in a cavity of the prostate gland, where, from the orifice which first admitted it having contracted, or the size of the calculus having enlarged, the stone could not be pressed back into the urethra, and the whole of the prostate gland had been changed into a capsule surrounding it."

I possess a preparation in my museum with cysts exterior to the urinary bladder, one of which may hold from four to five ounces. These communicate with the bladder. Another preparation where the right lobe of the prostate gland forms one capsule.

I confess freely that I was not prepared for the complication just described, nor am I ashamed to confess it, since no mention is made of such an anomaly in the writings of the most eminent surgeons, if we except Crosse and Wilson, from whose works I have quoted above, but which I had not seen.

JOHN LIZARS.

*Edinb.* 38, *York-place,*
21*st Jan.* 1836.

---

ST. THOMAS'S HOSPITAL REPORTS. By JOHN F. SOUTH, Assistant-Surg. No. 1, November, 1835, pp. 118. Sherwood and Co.

We rejoice to see that one of the largest, and certainly the most ancient, hospitals in this metropolis, has come to the determination to publish the reports of cases, both medical and surgical, in an authentic form, and under the superintendence of a responsible editor. This plan, upon the whole, must ensure the greatest proportion of good, attended probably with the least modicum of evil. The WHOLE TRUTH may not always be told in each particular case, or perhaps some cases may be occasionally (prudently) omitted—but still the public is secured against false statements, or statements too highly coloured by friendship or enmity. Guy's Hospital follows the example of its neighbour, and has published its first Number on the 1st January last. We shall make our readers acquainted with both reports in our present Number.

MEDICAL REPORT 1.—DROPSY.

"*Dropsy terminating in profuse Diuresis.** William Crane, æt. 33, coal-porter, admitted into Jacob's Ward, August 27th, 1835, under Dr. Burton. Of large frame, but with pale and flabby fibre, is the subject of hepatitis, ascites, and anasarca. It cannot be ascertained whether there be any enlargement of the liver, on account of the swollen state of the abdomen, but from the symptoms there can be no doubt, at least, of functional derangement of that organ. Respiration is free, and there is no sign of organic disease of the heart. He has not any pain in the loins, nor deposition in the urine. The absorbent glands of the neck are greatly enlarged, forming a kind of collar, which extends from ear to ear. The thighs and legs are much swollen, and the latter are in a state of considerable diffused inflammation, with dirty-looking ulcers on them, around which are scattered pustules; these he ascribes to the use of a wash he had applied when under treatment in a hospital, from which he was discharged about three weeks since. He is able to walk about, and does not complain of pain in the cavities, except when pressed on the region of the liver. He was put on milk diet, and ordered *Hyd. subm. Pulv. digit. Pulv. scill.* āā gr. j. *ter die. Infric. Ung. iod. abdomini.*

Aug. 31. *Ung. iod. mit. gland. cervic. infricand.*

Sept. 2. The medicine having acted rather sharply on the bowels, it was discontinued, and the following prescribed.—*Pil. hydr.* gr. ijss. *Pil. scill.* gr. ij. *omni nocte. Tinct. scill.* ♏xx. *Sp. æth. nit.* ʒj. *Dec. tarax.* ℥ij. *ter die.* This was continued till

Sept. 16th, When, in its place, was ordered *Liq. Potass. hydriod. (Lugoli)* ʒij. *ex Dec. tarax.* ℥iv. *ter die.* This was continued without any change occurring in his condition till

Sept. 20. On this morning he was suddenly attacked with vomiting and cold shiverings, succeeded by great heat of skin and profuse perspiration, and, when visited by the apothecary, the shiverings, heat of skin, and perspiration were found co-existing—the respiration natural as to frequency, but very laborious, and attended with loud, grating, sibilous and mucous rattle. The inflammation of the left leg had extended up and along the inner side of the thigh, and presented a livid appearance; an enlarged gland, also, on the anterior edge of the left arm-pit had become very red and painful. He makes no complaint, but, when asked, says he has some pain in the chest; and he answers questions hesitatingly, which is not his usual manner. There is neither pain in the head, nor greater heat on it than that of the general surface—there is no injection of the conjunctivæ, and the pupils are not dilated, but contract on the approach of light. He vomits frequently—bowels have been twice moved. Within the last two hours, he has passed about a quart of urine, exhibiting the appearance of

* Reported by Mr. Stone.

chalk and water. *Omitt. medicam. præced.* ℞. *Tinct. opii,* ♏xxv. *ex Mist. camph. stat., et Mist. effervesc. 2dà vel 3tiâ quâque hor. si urgeat vomitus. VS. ad* ℥xvj.

*Evening.* Breathing considerably relieved, and he answers questions with less hesitation. He has passed urine in excessive quantity throughout the whole day, amounting to a common-sized pailful and a half, which has the same appearance of dirty chalk and water. For about five minutes after the urine is passed, it remains perfectly clear, but then becomes gradually turbid. He has had several very liquid dejections, of the same dirty chalky character, but containing some bile; and he has vomited several times throughout the day, matter of the same character and appearance. The blood is much buffed, cupped, and so firm as to retain the impression of the finger, which is with some difficulty forced through the coagulum. He expresses himself better.

Sept. 22, *Morning.* He continues passing urine in still larger quantity, a great portion of which has been thrown away, but there is now a pailful standing by the bedside, of the same kind as that already described. The vomiting and purging also still continue. Breathing has again become more laborious. The perspiration and heat of skin are excessive. He appears partially soporose, but the pupils act freely, and he is quite conscious. Pulse about 100, full and firm. *VS. ad* ℥xij. *Addant. Acid. Hydrocyan.* ♏ij. *Tinct. op.* ♏viij. *dos. sing. Mist. efferv. Cat. sinap. epigastrio.*

The vomiting and purging continued throughout the day, and the urine was still passed in immense quantity. Towards evening the perspiration became cold and clammy—the pulse smaller and quicker—the breathing more difficult—countenance anxious—he became restless and unconscious, and expired about 7, P.M. The anasarca of the thighs and legs was somewhat diminished, but the abdomen retained its fulness. Sister states that this morning she noticed the swellings in his neck had considerably subsided since yesterday, and towards evening his neck had become quite thin.

The friends would not allow an inspection.

---

The following is the report of the chemical examination of the urine, by Mr. Sandell:

The urine, containing an immense deposit, was of a yellowish-white colour, very acid, and having a specific gravity of 10·47, but some voided the same day was only 10·38 at 60° Fahr.

Filtered, and the *urine* tested for albumen gave only a trace."

"There were then in the sediment—

| | |
|---|---|
| Animal matter, | Magnesia, |
| Ammonia, | Phosphoric acid, |
| Soda, | Muriatic acid, |
| Lime, | Lithic acid." |
| Potash, | |

### Report 2.—Delirium Tremens.*

"Henry Grey, æt. 39, admitted into Luke's Ward under Dr. Roots.

Aug. 9, 1835. A man above the ordinary stature, and of rather robust habit. From his mother's account, it appears that, for the last twenty years, he has been subject to fits, in which he falls down suddenly, perfectly insensible, with convulsive movements of his limbs, and remains in this state for a few minutes, or, at the most, for a quarter of an hour. He does not bite his tongue nor foam at the mouth, but, after the fits, discharges large quantities of flatus from the stomach. It is also stated, that occasionally he does not fall down, but walks some distance perfectly insensible; and that since the occurrence of a very bad fit twenty years ago, he has dragged his right leg after him, both, however, being weak. About three years and a half ago, after being in a state of intoxication for three successive days, he had an attack similar to the one about to be described, but more severe. He has lately been in bad circumstances, which appear to have acted very much upon his feelings. On

---

* Reported by Mr. Gibson.

Thursday (August 6th), he took a bottle of wine; on Friday, he was not perfectly rational; on Saturday fell down in a fit, and from that time his delirium has been on the increase.

When admitted into the Hospital his skin was hot—forehead bathed in perspiration—continually delirious, talking incoherently, and repeatedly uttering the same words over and over again. His face was flushed—eyes brilliant—pupils rather contracted, and contract still more at the presence of light. Countenance not much altered from the natural state, but frequently animated by a laugh at his own jokes. He says he feels no pain in his head or elsewhere, except in his feet, which, by the medical man's direction, previous to his coming into the Hospital, were to have been placed in warm water, but, by the want of care of his attendants, were immersed in very hot water, which completely blistered the greater part of them. All his toes have some portion of the cuticle separated from them, together with large blebs on other parts, and he seems fully aware of their condition. His tongue he puts out after a little solicitation; it is very slightly coated, and rather dry. There is no tenderness of his abdomen, but it is rather tumid at the lower part, and he discharges much flatus per anum. His bowels were operated on by medicine before his admission, and he was likewise bled twice, and had leeches on his head. Pulse 120, full and admitting of pressure. *V.S. ad* ℥xvj. *Lot. frigida capiti.* ℞. *Hydrarg. submur.* grs. iij. 6*tis horis.*

Aug. 10. He passed a very restless night, was delirious and raving at intervals. His head and skin are very hot, and bedewed with copious perspiration. He complains only of his feet, and strongly entreats every person who comes near him to loosen the straps by which he is confined to the bed. It should have been stated that he was brought to the Hospital in a straight-jacket, and has remained in one ever since, and has also been fastened to the bed by leather straps. Tongue is clean and a little moist; pulse 120, rather jerking.

*Evening.* He continues much the same, has been raving and talking indecently and loudly all the day. *C C. ad* ℥viij. *occipiti. Glacies capiti raso applicanda.* ℞. *Morph. mur.* gr. ss. *Hyd. subm.* gr. iij. 4*tis horis.*

Aug. 11. For the first hours of the night he had only a few seconds' sleep, and would then awake, and rave most furiously, but towards morning he had three hours and a half's rest, and when he awoke appeared better. He is now much quieter, is more rational, appears to know some of the events of the preceding day, still, however, wanders a little, but the furious delirium has quite gone off; he became tranquil after taking the two first doses of morphia. His skin is hot—face not so much flushed—pupils rather contracted in the dark. He says he has a little pain in his head. Pulse 116, full and soft; tongue clean and moist; bowels open. *C C. ad* ℥viij *occipiti.*

Aug. 12. He passed a pretty good night; slept very well, but wandered at intervals. He is now sensible—answers quite correctly, but at times talks incoherently. His skin is quite cool—countenance not flushed—pupils contracted. He says he has no pain in his head, but feels drowsy and languid. His hand trembles a little when extended—tongue clean and moist—bowels open—pulse 86, rather full. To be released from confinement. *Sumant. hyd. subm. et Morph. mur.* 6*tis horis.*

Aug. 13. He did not pass quite so good a night; there is more heat of skin to-day and more general excitement—he wanders a little at intervals, but is nevertheless very quiet—countenance rather flushed—pupils very contracted—no pain in his head—mouth sore—tongue clean and moist—bowels relaxed—pulse 88, fuller, and having some jerk about it. Feet very painful. *Omitt. Hydr. subm. Cat. pan. pedibus applicand.*

Aug. 14. Passed a very good night, but continues much in the same state. Pulse 82, not so full, but somewhat jerking, *Jusc. bov.* Oj. *quotidie. Morph. mur.* gr. ss. 8*vis horis. Cat. pan.* c̄ *Lot. plumbi, pedibus.*

Aug. 15. Passed a very bad night, and was very restless, tossing about his bed, and talking very loudly and incoherently, and becoming so violent as to compel the attendants to put him under confinement again. Skin rather hot—face slightly flushed—pupils contracted. He took this morning *Morph. mur.* gr. j. after which he had some sleep, and is now pretty quiet. Tongue moist and clean—bowels open four times during the night and once this morning—pulse 94, rather fuller and jerking. *Morph. muriat.* gr. ss. 4*tis horis.*

Aug. 16. Passed a very good night and is quiet to-day, but he wanders a little occasionally. Face not flushed, but pupils still contracted—tongue moist and clean—bowels relaxed—pulse 90. Feet very sore, but not quite so painful. ℞. *Mist. cret. comp.* ℥jss. *post. sing. sed. liq. Applic. Ung. zinci pedibus.* To be again liberated."

From this time the man gradually recovered. Dr. Roots has appended a valuable running commentary on this case—and on delirium tremens generally, which is deserving of careful perusal.

---

### Lectures on Medical Statistics. By Robert Graves, M.D.

We extract the following observations from one of a series of excellent clinical lectures, delivered by Dr. Robert Graves, and published in our contemporary, the London Medical and Surgical Journal.

### *Chances of Life.*

As a general fact it may be stated and admitted, that of a given number of children born in this country, one-third die before one year, one-half before the eighth year, two-thirds before the thirty-eighth, and three-fourths before the fiftieth year. But such general facts are drawn from data in the mass, and must necessarily include facts which differ toto cœlo from each other ; for averages of course include opposite extremes in their expression. Dr. Graves relates two interesting opposing facts.

"On Patrick's day, in the year 1782, nine students walked from Trinity College, Dublin, to Bullock, where they dined. In 1833 eight of them were still alive, and I have not heard of the death of any of them up to the present period. I mentioned this fact the other day to a very fine old gentleman aged seventy-six, and he observed that he could communicate another of an opposite description. He said that he had served in the Irish volunteers in 1782, and that of his entire grenadier company, consisting of fifty men, there was only one now living. What a different fate overhung these two groups of persons. It is remarkable how long a certain set of men holding the same office sometimes happen to live in succession.—There can be little doubt that the steady policy of the imperial court at Vienna has been partly, at least, owing to the extraordinary length of time the modern prime ministers of Austria, including Metternich, have lived. A similar successive longevity on the part of the rulers of Prussia, has aided other circumstances in aggrandising their territories, as has been well remarked by a late writer in the *Edinburgh Review.*"

Dr. Graves mentions the following as a calculation of the chances of life approaching to the truth. The chances are equal that every healthy adult will live half the difference between his own life and eighty-one. This, which is the mode of computation generally employed, is very near the truth. Thus, if a man be forty years of age, the chances are equal that he will live half the difference between it and eighty-one years, viz. twenty years and a half, and, therefore, that the duration of his whole life will be about sixty years.

Our readers are aware that the total numbers of male and female children brought into the world are very nearly equal, a small excess existing on the side of the males. In Germany and England the proportion of males to females is as twenty-one to twenty, while in Ireland it is generally as twenty-one to nineteen. But this slight excess is ultimately corrected by the greater mortality of the males before the period of puberty. After that age the excess is slightly on the female side.

Dr. Graves relates some interesting observations of M. Giron's, with regard to the relative numbers of males and females born from a given number of marriages in different ranks of life. M. Giron divides individuals into different classes; the first is composed of individuals whose employments tend to develop their bodily powers; the second, of those whose business tends to enervate; the third, of those whose employments are of a mixed description. He found that, in the first class, the number of male births exceeded the average proportion of male and female births throughout France: that, in the second class, the number of female births, exceeded the average proportion of female to male births throughout France; and that, in the third class, the proportion of male and female births was nearly the same as the average proportion throughout France. Hence he arrives at the conclusion, that the pursuits of agriculture tend to the increase of the male population, and that the habits of commerce and manufactures favour an augmentation of the female population."

The next point alluded to by Dr. Graves is the variety in the proportion of births to a marriage.

"It has been supposed," says he, "that the fecundity of marriages is in proportion to the comfort and independence of the community, and that fewer children are born from a given number of marriages in countries which are deficient in agriculture, industry, and the blessings of civil liberty. This, however, is not the fact. In England the proportion of births to marriages from 1800 to 1810, was as 4 to 1, and from 1810 to 1821, as 4·22 to 1. In Scotland and Holland it is as 4·2 and 4·20, while in Russia and several of the Italian states it is as 5·25 and 5·45, to 1. I shall not take up your time with these statistical details any farther than to observe, that in the degree in which a nation advances in prosperity and civilisation, premature and imprudent marriages will become less frequent, and the number of births be proportionally diminished. The lateness of marriages may be generally taken as a good test of an improved state of society, as exhibiting that power of moral restraint over his passions which should characterise civilised and intelligent man."

We fear the latter observations is more characterized by charity and good feeling, than by absolute truth. We greatly doubt if morality increases with civilization, or if late marriages be a proof of restraint over the passions. In marriage, woman is the passive agent, and men decide its period. Necessity, in a country like our's, prevents the early marriage of persons in the middle, and perhaps, also, in the lower class. The men will not marry and the women cannot. The latter are compelled to restrain their passions, if any such are felt, but are we so certain that the same restraint is practised by the former? Do the countless cyprians that throng our streets, and flaunt in every public spot furnish unexceptionable evidence, of the continence and moral rectitude of man? We fear that they do not.

*Lives of Literary Persons.*

"I had almost forgotten to mention the remarkable fact, that the cultivation of science and literature appears to be favourable to longevity, contrary to the opinion generally entertained on this point; indeed it seems that the man who is chiefly engaged in mental labour has a fairer prospect of length of years than he whose occupations consist in exclusive bodily toil. Franchini has enumerated one hundred and four Italian mathematicians of different epochs; he has ascertained the ages at which seventy of these died, and among them we find eighteen who had attained the age of eighty, and two of ninety, and this in a climate which is not generally considered to be favourable to longevity. In France, one hundred and fifty-two men of science and letters have been taken at random; half the number appears to have cultivated science, and about half to have devoted themselves to general literature; on adding together the age at which each of them had died, it was found that the average result would be above sixty-nine years, for each individual.*

*Comparative Longevity of Female Authors of the last Century—Quarterly Review, No. 99, on Madden's Infirmities of Genius.*

| | Age | | Age |
|---|---|---|---|
| Lady Russel .. | 87 | Mrs. Chapone.. | 75 |
| Mrs. Rowe.... | 63 | Mrs. Lennox .. | 84 |
| Lady M.W. Montague ...... | 73 | Mrs. Trimmer.. | 69 |
| Mrs. Centlivre.. | 44 | Mrs. Hamilton . | 65 |
| Lady Hervey .. | 70 | Mrs. Radcliffe.. | 60 |
| Lady Suffolk .. | 79 | Mrs. Barbauld . | 83 |
| Mrs. Sheridan.. | 47 | Mrs. Delany .. | 93 |
| Mrs. Cowley .. | 66 | Mrs. Inchbald . | 68 |
| Mrs. Macaulay. | 53 | Mrs. Piozzi .... | 80 |
| Mrs. Montagu . | 81 | Mrs. H. More.. | 89 |

*Suicides.*

Dr. Graves mentions facts connected with suicides not devoid of interest.

Suicides are not so much the privilege of John Bull, as some persons suppose. There are more in France than in England, indeed it is probable that for one in London there are five in Paris. Irritation is a frequent cause of suicide, and of this our author mentions several instances. Industrious and prudent people very seldom commit suicide. Out of 120,000, who insured their lives in the Equitable Insurance Office, the number of suicides in twenty years was only fifteen.

The Irish are the least suicidal nation in Europe. Dublin and Naples are the two cities in which fewest suicides occur. Yet in both the poorer class are very poor indeed. Dr. Graves observes that an Irishman often murders his neighbour but never dreams of killing himself. The fact is that the prevalence of murder prevents the necessity for suicide. If both prevailed the "seven millions" would be reduced. There were forty murders in Ireland for one committed in Prussia, and in Prussia there were forty suicides for one committed in Ireland. There *were*, for the calculation was made ten years ago. Both nations have since materially changed. Fewer Prussians, in proportion, are now killed by themselves, and many more Irishmen are now killed by others.*

The Lectures of Dr. Graves, from one of which we have drawn the preceding facts, are well worthy the attention of all who take an interest in medical science.

* This average duration of the lives of literary men which is given by Hawkins, is evidently greater than can be allowed, neither are Madden's tables of the mean duration of the lives of *literati* free from the same error, as is well shown in the *Quarterly*.

* If official statements are to be relied on, the increase of murders in Ireland has been progressive since the *penultimate* rebellion.

---

ON THE MEDICINAL PROPERTIES OF CREOSOTE. By JOHN ELLIOTSON, M.D. F.R.S. &c.

As pathology discovers, or seems to discover, that some diseases are, if not incurable, very like it, so it fortunately happens that the world has generally before it some remedy, which cures or relieves what nothing would cure or relieve before it. It is true that a little time dispels the pleasing dream, and we find, after all, that our stock of specifics remain as they were.

Dr. Elliotson is, perhaps, as indefatigable an experimentalist as any physician in London. Did we "hint a fault," we should say he was too sanguine. His present researches on creosote, will, we hope, be attended with beneficial results. The paper containing them is in the last volume of the Royal Medico-Chirurgical Society's Transactions. Phthisis, epilepsy, neuralgia, Asiatic cholera, vomiting of different sorts, diabetes mellitus, ulcers of the skin, hiccup, acné indurata, chronic glanders, are the maladies in which our author has given it with more or less success in each instance. The diseases *appear* to be so opposite, that reason would hint the inapplicability of any one remedy to all. But reason is frail, and facts are stubborn.

1. *Phthisis.* "My trials with it internally in phthisis were perfectly unsuccessful. I have since made many phthisical patients breathe for four or five minutes, four or five times a day, through a mixture of it with mucilage and water, but with no further effect, in general, than occasionally an increased facility of respiration, and a diminution of the cough and expectoration. I put one drop into rather less than a pint of cold water, and add one drop every time it is employed, in order to maintain the strength of the liquid, till this appears growing too strong; and then the patients inhale without adding any, till the liquid appears growing weak again. Some it always appears to irritate, and all in whom any degree of inflammation exists. I am satisfied that it is no remedy for tubercles. Where, however, only a single ulcer, or but a small number exist in the lungs, and there is no disposition to farther tubercular formation, it is very beneficial. One young gentleman, with a large solitary cavity in his left lung, has completely recovered, and not the slightest morbid condition is discoverable by the ear. In bronchorrhœa, or that state of the bronchial mucous membrane which consists in a profuse secretion without inflammation, I have seen its inhalation of essential service. In one instance of this affection, in which the expectoration was extremely offensive, the cure was very rapid. In asthma, also dependent upon morbid excitability of the bronchial membrane, its inhalation is often useful."

Dr. Elliotson affirms, that creosote has been useful where "only a single ulcer, or but a small number, exist in the lungs, and there is no disposition to further tubercular formation." In the first place, we should be anxious to learn how such an accurate diagnosis as the determination of a single ulcer in the lung is made. We believe we are not quite unpractised in auscultation, but to such accuracy of diagnosis we cannot pretend. In the next place, we would observe that a single ulcer, or a few, without any disposition to farther tubercular formation, is any thing but common. Such cases are not often met with. Crude tubercles are usually widely scattered, at all events over the superior lobes, prior to ulceration of the most advanced—or tubercular infiltration has made progress prior to the same sequela. Dr. Elliotson has not been experimenting long with creosote. It is probable that further experience will induce this talented observer to qualify the very favourable opinion which he now entertains of creosote.

2. *Epilepsy.* In this, creosote did such negative good, that it may be said to have done nothing.

3. *Neuralgia.* "The first case of neuralgia, or what appeared so, occurred in a girl, 12 years of age, who, after the influenza a year previously, had gradually become so costive, as to have an evacuation but once in three or four days, and then with great pain. In this condition she was suddenly seized with spasms in the abdomen, twitchings in the legs and arms, and extreme agony in the lowest part of the abdomen and pelvis. An attack of this kind frequently recurred, and at length came on every morning, about 7 or 8 o'clock, and lasted till night, when she fell into a comatose state till towards morning. The pain was such that she constantly sat moving backwards and forwards, wringing her hands, or pressing the lowest part of her abdomen; her face was expressive of intense suffering, and she was much reduced. At the time of her admission into the hospital, she had made water but once in twenty-four hours for the last three months, the abdomen was very tense, though but little swollen, and gave a hollow sound on percussion. The urine and alvine discharge were of a healthy character, and the bladder on sounding gave no indication of disease. Every known medicine likely to prove beneficial had been exhibited in the country. She was one of the most distressing objects I ever beheld, and I utterly despaired of any improvement. Three grains of muriate of morphia every morning perhaps alleviated her sufferings; but so little, that I discontinued it. On the 22d of July, I ordered a drop of Creosote to be taken three times a-day, and the dose was gradually increased to seven. She began to improve rapidly, and left the hospital perfectly well in a month from the commencement of its use; and in the mean time she had regained her flesh and every appearance of perfect health."

The next case was one of neuralgia of the posterior dental and nasal twigs of the superior maxillary nerve. The pain had existed for three months. On a former occasion, a similar attack had passed away without any medical assistance, and it is not, therefore, greatly to be wondered at that it went away this time, whilst creosote was taken. In two other cases, the remedy nearly subdued the complaint, but Dr. Elliotson adds that he has frequently known it to fail. Of this we have no doubt, nay, we venture to prophecy that the oftener it is tried, the oftener it will be found useless. Dr. Elliotson adds, that the "rare and strange forms of hysteria have yielded to creosote." We should be very loth to expect such good fortune in our own experiments. The rare and strange forms of hysteria go as they come, without our being enabled to determine how or why. He who looks to do much in such cases with physic, and particularly with empirical drugs, will find himself wofully mistaken.

4. *Vomiting.* The power of creosote in arresting vomiting, is considered by our author as perfectly established.

"When inflammation of the organ exists, the stimulant power of the medicine must do harm, and more than counterbalance its soothing properties. Where structural disease exists in the stomach, the diseased surface may not bear such a stimulant except in the minutest quantity; and a minute quantity, even should it arrest the vomiting, is very likely to aggravate pain. Although some do not dislike it, and others who dislike it at first actually find it at length rather agreeable, a few are so disgusted with the smell of tar, that they are made sick by any attempt to take Creosote: but, with these exceptions, I know of no medicine at all to be compared with it in arresting vomiting. I have repeatedly seen it succeed after the failure of prussic acid, which is the most powerful remedy I previously was acquainted with in subduing this action of the stomach. Different doses and different frequency of repetition are requisite to produce this effect in different instances. The dose may easily be too large or too small. More than two drops I have sometimes seen aggravate the sickness, and sometimes I have begun with three drops every three hours, and been obliged even to give more. One or two drops may be given every hour or half hour till the vomiting ceases; and, if a dose is rejected, it should be repeated immediately. The first dose frequently succeeds. I could detail fifty cases of vomiting in the practice of myself and friends, and both in public institutions and in private practice, illustrative of its extraordinary power in this respect. In colic and enteritis it arrests the vomiting long before the bowels are opened, and purgatives are thus retained which were all rejected previously to its exhibition. Even in a case of severe vomiting, apparently from arsenic, which usually excites inflammation of the stomach, I have known it succeed astonishingly, as well as in the only case of vomiting from pregnancy in which I have had an opportunity of trying it: and in sea-sickness, in which, however, my experience of its power is yet limited, though a number of my friends have promised to take it abroad with them. Of course, as it subdues vomiting, its power is equally great over nausea. When *properly* given in nausea or vomiting, without inflammation or structural disease of the stomach, I have not yet known it fail, except in one remarkable case.—A boy, fourteen years of age, had, for two years, instantly vomited whatever he took, without effort and without any diseased condition with which the occurrence could be connected. He was rather weak, thin, and pale, but otherwise in perfect health, except that he confessed being rather giddy, and had a habit of knitting his brows,—symptoms which at length made me suspect that the cause was in his head. Creosote, as far as it was tried, failed in him, though given in doses of three drops and repeated frequently, so frequently in one day as thirty times. I proposed augmenting the dose, but he was unhappy at being from home, and left the Hospital of the University."

Dyspepsia has been treated by our author with creosote, and when it fails he unites with it prussic acid. Dyspepsia is but a name, implying disordered functions of the organs of digestion. Generally speaking, that disorder is either morbidly-excited sensibility, or a slight degree of inflammatory action of the mucous membrane. One or other of these states is the essential condition of ninety-nine cases out of every hundred of indigestion. Is a stimulating drug likely to cure that, or to increase it? This we know—that treatment precisely the opposite *in principle* is what agrees the best, and this is sufficient for us.

5. *Diabetes.* "On the 13th of August, I was requested to see a gentleman from the country, about sixty years of age, plethoric, with a full pulse: his mind was dull, and he had suffered two attacks of paralysis: his tongue was very yellow, and black at the centre towards the root. He told me his complaint was extreme thirst, so that he was drinking all day, and was enraged if drink were not taken to him the moment he called for it. He said he had been ill four or five years, was much worse always in autumn, much better in spring. He passed but four pints of urine, according to his own account, but confessed that he made water twelve times a day and three times in the night. I found it contain a large quantity of sugar. I ordered him Creosote. I saw him again on the 10th of September, when he was making water but six times in the day and once in the night. It contained scarcely any sugar; his tongue was clean, and he told me that he felt perfectly well in every respect. The qualities of the urine I could not ascertain."

In the next case, that of a young medical gentleman, the improvement was surprising. But—he was not cured, for two months ago he was seen, only no worse.

In the next case the patient went abroad, consulted many persons, neglected his medicine (a pretty good proof that it did him no good) and—died. In short, we may safely add creosote to the list of remedies that will—*not* cure diabetes.

6. *Acne indurata.* Creosote continued for six months accomplished all but a cure.

7. *Chronic Glanders.* "But I am anxious to mention its effect in two cases of chronic glanders, affecting one nostril and the frontal sinuses with pain and a copious and fetid discharge. The disease in the two persons was clearly contracted from a glandered horse, and I purpose doing myself the honour of laying the facts before the Society early next session, as I never read of or met with an instance like these in the human subject; former cases having been acute glanders, or chronic farcy. The sedulous injection of a weak solution of Creosote up the nostril, removed the whole of the symptoms, after a very few weeks, and I hear the patients are still well."

Such is our able author's experience with creosote. We are not unsanguine in these matters. A reviewer sees the rise and fall of new drugs, and each has evidence at first in its favour. But the fiat of the mass of the profession seldom confirms the flattering anticipations drawn from early trials.

P.S. Dr. Roots, the talented physician of St. Thomas's Hospital, has recently given a clinical lecture at that institution, from which we make the following extract, as pertinent on the present occasion.

"I perfectly agree with Dr. Elliotson, that creosote is often of considerable value in allaying irritability of the stomach, when perfectly independent of any inflammatory action. I am disposed, on the whole, to say that creosote is entitled to hold the same rank in the materia medica, as a remedy in irritable conditions of the stomach, free from all inflammatory action, as does the OXIDE OF BISMUTH. After having used it from the first moment of its introduction in this country, I have not been able to do more with creosote, in irritation of the stomach, than I have with the oxide of bismuth."*

---

### TUBERCULAR DEVELOPMENTS IN THE MONKEY.

[Extract of a Letter from Mr. A. Dickson, of Rachan, in Scotland.]

I may mention here, that I had lately an opportunity of dissecting a *monkey* which died of phthisis, and which exhibited the finest specimen of tubercular disease I have ever seen. It admirably illustrated some of the general conclusions established by Louis, from his extensive researches in this department of pathology, which were ably epitomized in one of your late Numbers. The fact, that when tubercles are co-existent in the lungs and in other organs, they are invariably most developed in the former, while their development in the latter is in general equal, was most strikingly verified. The liver and spleen both contained tubercles, which shewed no signs of suppuration, while, in the lungs, suppuration had proceeded to a great extent. The pulmonary tissue, especially in the left lung, (which supports another of Louis' conclusions) was almost completely annihilated, and its place occupied by the tuberculous deposit. The left lung was so firmly attached to the walls of the thorax, in almost its whole extent, that it could not be detached by the most careful dissection, but required to be sliced away. The suppuration at one point, instead of finding its way into the bronchial tubes, had penetrated the intercostal muscles, and formed a tumour externally, the size of a pigeon's egg. The preparation is in the museum of the Royal College of Surgeons, Edinburgh.

A. D.

* Dr. Ryan's Journal, Jan. 23rd, 1836.

---

### FLANNEL IN HOT CLIMATES.

In the United Service Journal for Aug. 1835, there is a very valuable paper by Dr. Ferguson, on the "Health of Troops," which we strongly recommend to our military medical readers—and, indeed, to all military men, whether medical or not.

We were gratified to find our own opinions, respecting the use of flannel next the skin in tropical climates, corroborated by a talented army physician. Dr. F. observes :—" I, for one, protest against it (flannel) as an enervating habit, of which the healthy, hardy soldier can never stand in need. To the feeble and valetudinary it is most useful; and, as an hospital indulgence, highly proper; but, when worn in the crowded barrack-room, with, too often, bad washing and insufficient change, it becomes a deposit of filth—even of contagion, irritating to the skin, and incompatible with health and cleanliness." Dr. F. very properly makes some exceptions, such as in bivouacs, and when the soldier is encamped, and in a rigorous climate. " With the above exceptions, it should never be seen either in the barracks or quarters."

---

### TRANSCENDENTALISMS.

Our exquisite, as well as excellent contemporary—the BRITISH AND FOREIGN —has opened a splendid BAZAAR of Continental commodities, of the latest fashion, and so recherché, that all other bazaars of this kind may shut up shop at once! We are very far from casting an eye of envy or jealousy on this neighbouring REPERTORIUM :—On the contrary, we welcome the STRANGER with the most cordial greeting. Our's is a warehouse of plain goods—chiefly of British manufacture—and containing only a moderate assortment of such foreign articles as we judge suitable for home consumption and ready sale. We are delighted, then, that a "PUBLIC LOUNGE," in the medical market, is opened, such as we see in the Strand and Holborn, for those dandies who relish exquisite morçeaus, and like not the coarse, though wholesome, provender adapted to the generality of English palates. So far from entertaining the petty feelings of mean rivalry, and cautioning our customers to avoid the "BRITISH AND FOREIGN" Bazaar, we shall always be happy to direct their attention to the shop in question, as one where they will be sure to find the most piquant specimens of transcendental medico-chirurgical spicery. Nay, we will go farther. We will purchase from our neighbour—though he disdains to purchase from us—and always keep "on sale or return" (to use a Paternoster phrase) a small (but select) assortment of transcendentals, for the benefit of trade, and the mutual advantage of both parties.

In another place, we have quoted a most remarkable "Russian puzzle," which will supply materials for conjecture to a great number of British exquisites. In this place, we shall quote a short extract or two, from some experiments by the illustrious TIEDEMANN, on "PULMONARY EXHALATION," though we candidly confess that we see little probability of their conducing much to practical utility—the real and the *avowed* object of this Journal. The following passage will damp the ardent expectations of those who expect to arrive at accurate conclusions on the subject of pulmonary exhalation.

"Having mentioned the various results of experiments by different physiologists to ascertain the quantity of water given off by the lungs in twenty-four hours, Professor Tiedemann observes, that it is nearly impossible to decide upon the actual quantity of expired vapour, on account of the rapid absorption by the lungs, not only of air containing vapour, but also of vapour which had been already disengaged. The bulk and area of the lungs also varies exceedingly. The condition of the atmospheric air, its density, temperature, and hygrometric state, will doubtless have a considerable influence. The quantity of vapour thrown off by the lungs varies according to the condition of the body: in plethoric healthy individuals, a much larger quantity is formed than in those who are emaciated and weakly. Jurine, Lavoisier, and Seguin found that more water and carbonic acid were formed after meals than when the stomach was empty. Magendie observed, in a dog, after he had injected a considerable quantity of warm water into a vein, that the respiration was accelerated, and that a large quantity of water flowed from the mouth. It has also been shewn by the experiments of Prout and Fife, that much more watery vapour and carbonic acid are formed when a person is awake than when asleep: bodily exertion and mental excitement also increase it. Nysten found that in chronic diseases without febrile excitement, and where the lungs were healthy and the respiration natural, no change was observed; but that, in acute forms, the expired air generally contained more watery vapour and carbonic acid. Jurine found, in a case of ague, that less carbonic acid was disengaged from the lungs during the cold, than during the hot and sweating stages; in a patient who had been bled to sixteen ounces, less carbonic acid was found in the expired air after the venesection than before. Nysten observed that, in all diseases attended with dyspnœa, the carbonic acid and watery vapour of the lungs were diminished."*

Whoever peruses the foregoing extract will be a sanguine physiologist indeed, if he expects that anything determinate can be collected from experiments on this point. There can be no doubt, however, that substances taken into the stomach or rectum are soon discoverable (if they have any peculiar odour, &c.) in the expired breath. In order to ascertain what substances pass in the form of vapour from the blood in the lungs, TIEDEMANN instituted a se-

* British and Foreign, No. I., p. 242.

ries of experiments on dogs, several of which are detailed in the pages of our new contemporary. These we need not quote. The following passage contains the pith of the paper.

"The function of the lungs does not only consist in effecting an important and absolutely necessary exchange between the component parts of the inspired air and those of the dark red venous blood mixed with chyle and lymph, during which the oxygen of the expired air combines with the blood, and the carbonic acid is thrown off, producing the bright red arterial blood; but, besides this, the lungs appear to act as a genuine excretory organ for the venous blood. Volatile unassimilable substances, which have been conveyed into the blood from the food, which cannot serve in forming arterial blood, and are capable of evaporation, are thrown off in the cells and bronchi of the lungs, and removed with the expired air. Thus, the lungs assist in preparing the arterial blood from the alimentary matters which have been digested and carried into the circulating system, and impart to them such combinations and properties as fit them for the office of reproduction, and render the blood conveyed to every organ capable of repairing those changes in the structure and organs of the animal body which have taken place in the performance of their various functions."

---

### Pathology of Rheumatism.

There is a curious coincidence between two physicians respecting rheumatism. Dr. Johnson's observations were published in No. 44 of this Journal, p. 460, April, 1835. M. Parise published his sentiments in July of the same year, in the Bulletin General. Both writers labour to separate acute rheumatism from chronic, considering the former as *arthritis*—the latter, as a peculiar affection, probably neuralgic, of the investing sheaths of muscular fibres—or perhaps of the neurilema of the nerves themselves. The authors do not coincide, however, on one point. M. Parise looks upon acute rheumatism, as differing in no respect from common inflammation of a joint—say that which would arise from the kick of a horse, or a strain. Dr. Johnson, on the other hand, maintains that acute rheumatism is closely allied in its nature to *gout*, affecting usually the same parts, and very often not to be distinguished from the latter disease.

---

### On the Pathology of Phlegmasia Dolens. By Professor Jennings.

The following extract will shew that the pathology of the disease is by no means settled. For our own parts, we are inclined to think that fatal cases, *resembling* the phlegmasia dolens of females, and which have been traced to phlebitis, are not the ordinary cases met with, and formerly described by so many authors of undoubted observation and discrimination. How is it that almost all the cases *proved* to be phlebitis die, while there is scarcely an instance on record, of the common phlegmasia dolens proving fatal?

"The pathology of phlegmasia dolens still remaining in an unsettled state, a few observations on this painful affection, accompanied with something additional to the common method of treatment, may be acceptable to the readers of the United States Medical and Surgical Journal.

The supposition that it is produced by a translation of the milk from the breasts to the affected limb, which was formerly entertained by many of the most distinguished French and German writers on the subject, is no longer thought to be worthy of respect.

Doctor White, of Manchester, considered the disease to be consequent on a peculiar pressure made by the child's head against the brim of the pelvis, in the time of parturition. This opinion, although far from being satisfactory, is nevertheless entitled to respect, since it was the first advance towards a theory which could merit attention.

Dr. Davis ascribed it to a remora of the blood, returning from the extremity of the affected limb, and a consequent inflammation of one or more large veins. This opinion is in accordance with the theory of Velpeau, &c. But

*Dr. Dewees has made it sufficiently evident that the disease is not phlebitis.*

Drs. Trye, Ferriar, and Hull, with much unanimity, considered the disease to be seated in 'the lymphatic glands.' It is probable they were looking towards the extreme capillary structure of the lymphatic system. They do not appear to have varied materially from the opinions of Drs. Denman and Latham.

Drs. Dewees, Houston and Eberle, seem to be very nearly in consent with the last-named authors. They believe 'the whole system of lymphatic vessels of the affected limb, is engorged and distended with lymph, at the same time that some effusion may occur in the subcutaneous and intermuscular texture.'

Dr. Houston says, 'it may occur primarily in one or more glands, or it may have its origin in a principal lymphatic trunk, and thence extend to the glands.' That a gland or lymphatic trunk may be bruised by the child's head in its passage through the pelvis, so as to set up this peculiar kind of inflammation;—That exposure to cold, or damp air, or putting on damp clothing, may produce a similar effect;—Also, that the absorption or transmission of some acrimonious matter, may serve to induce the disease;—That in every instance it is preceded by one or other of the foregoing incidents, as its exciting cause. That the disease exists primarily in the glands and their communicating trunks, he thinks is made certain by 'the uniform occurrence of pain and uneasiness about the passage of the round ligament in the groin, and down the inside of the thigh,' antecedent to the swelling, or other affection of the limb. That the color of the affected limb, proves an engorgement of the lymphatics; whereas, a congestion of the bloodvessels, as in ordinary inflammation, would assume a red and not a white color.

And that the state of the pulse and tongue,—the character of the fever, &c. proves the existence of an irritation, such as ought to be looked for, when there is great distention of the lymphatics throughout their utmost ramifications, making pressure upon the immensity of nervous fibrils, by which they are surrounded.

The manner of its termination is considered to favor this conclusion, since it is more uniform and less fatal than an equal extent of inflammation consequent upon a congestion of the bloodvessels; and it commences with a diminution of pain in the iliac and inguinal regions, shewing the seat of the disease to be in the lymphatic system, and especially in the conglobate glands."*

The remedy which Professor Jennings recommends, from experience, is strips of adhesive plaster, spread with mercurial ointment, over the whole of the limb affected. The success exceeded our author's most sanguine expectations, and he now employs it in every case that comes under his care. He covers the limb thus treated with neatly fitted pieces of oil-silk, and a bandage over all, to fit the dressings closely to the whole surface of the limb—but not so tight as to produce pain. He gives a daily aperient of calomel and ipecacuan. As soon as the mouth begins to shew the effects of mercury, the symptoms abate.

---

### Singular Case of Stercoraceous Vomiting during a period of eighteen months, caused by Adhesion of the Sigmoid Flexure to the Transverse Arch of the Colon, and this again to the Great Curvature of the Stomach, with Ulcerated Communication through all these Parts. By Adam Dickson, Esq. Surgeon, Rachan Mill, N.B.

Mrs. F. ætat. 38, ever since the birth of a child, about 18 years ago, had been in very delicate health, and subject to occasional cough and expectoration, after exposure to cold, with constant dysp-

* United States Medical and Surgical Journal, Oct. 1835.

nœa periodically exasperated. About six months before she came under Mr. Dickson's notice, she had had an attack of abdominal inflammation. The treatment was so far successful, that she had been restored to nearly her former state of delicate health, except that slight general tenderness of the abdomen still remained, with a constant, acute, and lancinating pain in the left iliac region. When first seen, it was on account of a recurrence of the symptoms of a general peritonitis, with severe aggravation of the local complaint. As the patient was feeble, and considerably emaciated, recourse to general blood-letting did not seem warranted, and Mr. D. trusted to leeching, blistering, fomentations, calomel and opium, and mild purgatives, which had the effect of speedily relieving the constitutional symptoms, and removing the tenderness of abdomen, but did not produce any marked effect on the pain, which had remained ever since the former attack. It was most acute in the left iliac fossa, and extended upwards, in the direction of the descending colon, into the left hypochondrium. There was no external swelling or fulness; but, upon careful manipulation, a small hard tumour could be felt in the situation of the sigmoid flexure, which was most acutely tender when pressed upon, and was the point from which she described the lancinating pains as proceeding. The bowels were at all times costive, and she required the constant use of pills to regulate them, as she uniformly experienced an aggravation of the pain when they were neglected. About three months after having first seen her, and after having, in the mean time, used leechings, blisters, ointment of tartrate of antimony, mercurial and iodic frictions, narcotics, &c. without making any impression on the disease, she had an attack of decidedly stercoraceous vomiting, by which she evacuated about three pints of a thickish fluid in the course of the day. The vomiting returned a week after, in nearly the same quantity, but more markedly stercoraceous. The evacuations *per anum*, had, notwithstanding the exhibition of laxatives and enemata, been very scanty during the time which intervened between the attacks of vomiting, as well as habitually before the first. When the fæces were evacuated without assistance of laxatives or lavements, their diameter was small, affording a presumption that organic contraction of the gut existed, in the locality indicated by the lancinating pain. After this period, the vomiting, in spite of every remedy that could be tried, continued to recur more frequently and the natural evacuations downwards to become more scanty, until about four months before death it occurred every day with great regularity; or, if it missed one day, a larger quantity was brought up next day. The peristaltic motion of the bowels, except of a small portion at the lower end, seemed to be completely inverted; for, when a purgative was exhibited, it never failed to act as a purgative, in the inverse direction, and always brought away, by the mouth, a larger quantity of liquid fæces than was vomited on ordinary occasions; while clysters produced very little, and often no effect at all. These vomitings were extremely painful, being always preceded by three or four hours of very severe sickness and violent headache—the disgusting fæcal taste was never absent from the mouth, and the tongue was constantly covered by a thick yellow fur. The pain continued to become worse—the patient got no sleep, and could take no nourishment—emaciation became extreme—at length the hands and feet swelled—diarrhœa took place, and she died in a week from its commencement, and eighteen months from the origin of the complaint. The vomiting continued to the last.

*Inspection.* On opening the abdomen, the products of inflammation were sufficiently obvious, in numerous adhesions, which were apparent at first sight; but the most remarkable feature was, the almost complete concealment of the small intestines, by the displacement of the stomach and transverse arch of the colon, which, intimately and inseparably connected with each other, were both drawn out of their natural situation, and firmly adherent to the sigmoid flexure. Upon making a careful and minute dissection of these parts, it was found that their connexions and modes of communication by morbid openings were highly curious, and perhaps unprecedented. There was first, a large ulcerated opening, with foul and ragged edges, in the great curvature of the stomach, communicating with the transverse colon, which was inseparably attached to it. A similar ulcerated opening, through the opposite wall of the colon, led directly into the commencement of the sigmoid flexure, which firmly adhered to the transverse arch, in the same manner as the arch adhered to the stomach—the whole three, stomach, arch, and sigmoid flexure, being closely matted together in one point, and forming a firm, hard mass, which had been the tumor felt during life in that situation. The scirrhous ulceration had thus destroyed four folds of intestine—the coats of the stomach—both walls of the arch, and one fold of the sigmoid flexure of the colon, and formed in this manner a patent communication, of not more than two inches in length, between the stomach and the lower part of the large intestines, and in consequence cut off and obstructed the whole tract of the alimentary canal above the arch of the colon.

The opening leading from the great curvature of the stomach into the transverse part of the colon, and from that into the sigmoid flexure, was wide, and admitted two fingers with ease; while the continuation of the colon, on either side of these adventitious openings, was contracted by bands of cartilaginous firmness, so as to admit only the point of the little finger. The small intestines, and the colon above the diseased point, contained a very considerable quantity of consistent, but not hardened fæces, which proved that the colon, though its tract was still open to the extent already mentioned, had not admitted the passage downwards, of any of the contained matters; and that the diarrhœa, which had existed before death, had taken place entirely from the inferior portion of the great gut. The pancreas was of very unusual size, but its structure was perfectly normal. The lungs presented fine specimens of vesicular emphysema, but there was no traces of diseases in any other organs of the abdomen or chest.

This case is curious, both for the complication of the organic lesions which it displays, and for the great length of time during which the inverted action of so large a portion of the intestinal canal existed. It is perhaps unprecedented, that the mouth should, for such a long period, be the emunctory of the whole tract of the intestines, except the small part below the sigmoid flexure.

The scirrhous ulceration had, Mr. D. imagines, commenced in the sigmoid flexure—the situation in which pain was first felt: this, when its coats were nearly penetrated, had become attached to the arch of the colon by adhesive inflammation, a protective process which we find so often taking place to prevent effusion into the cavity of the peritoneum. During all this time there was no vomiting; but as the diameter of the colon became contracted, and the coats of the stomach involved, vomiting took place at intervals, to relieve the upper part of the intestinal tube of the contained matters, which could not find a ready exit downwards. As the contraction of the colon proceeded, and the coats of the stomach became perforated, the irritability of the ulcerated surface, would combine with the diminished diameter of the bowel, to prevent the passage of the fæcal matter along its tract, when the inverted action became fully established as it was for the long period mentioned. This mode of progression, and this order of the involvement of parts, is the most probable, and that which agrees with and best explains the symptoms observed. The principal subject of curiosity is, the endurance of life under such extraordinary circumstances.

---

### Dr. Bostock on the Chemical Composition of Calcareous Tumors.

Dr. Bostock has lately been at the pains of analysing several calcareous tumors of the uterus, as well as calcareous depositions in other organs and parts of the body. The analysis of salivary and other calcareous concretions had previously informed us that they were usually composed of phosphate of lime, a small proportion of the carbonate, and a certain quantity of animal matter. We say usually, for M. Lassaigne, in an examination of a salivary calculus, found this order of composition reversed;—86 per cent. being carbonate of lime, and only 3 per cent. the phosphate.

Dr. Bostock has, through that indefatigable and excellent physician Dr. Robert Lee, had recently an opportunity of investigating the chemical composition of nine different calcareous growths, in addition to one obtained from Mr. Howship. We will specify the results of his investigations in each instance.

1. The first specimen consisted of calculi discharged from the uterus during life. They were made up of a number of small bodies, from the bulk of a pin's head to about three times that size; their form was completely irregular, and in some parts they appeared as if they had been corroded, resembling a decayed tooth. They were of a yellowish white colour, like bone or ivory, and were so hard as not to be cut with a knife. Some of them were single, while others were connected by a membranous substance: the membranous substance had a few dark brown bodies, of the size of a mustard seed, attached to it. The whole, when dried at the temperature of 200°, weighed 6·2 grs.

A portion of the substance was subjected to a red heat; it burned slowly, with a dull flame, and lost about one-fourth of its weight. The residue was treated with dilute muriatic acid, and, with the exception of a few black particles, was quickly dissolved. Another portion being digested in boiling water, lost neither weight nor form, and the fluid contained no appreciable quantity of albumen or of gelatine; but there was a minute portion of the animal matter which exists in the serosity of the blood, and a trace of common salt. The substance being again dried, was digested in dilute muriatic acid. The solution was found to contain a considerable quantity of phosphate of lime, with a small quantity of the carbonate. The dark brown particles formerly mentioned are supposed by Dr. Bostock to have been clots of blood. On being treated with caustic potass, a membranous film remained, which assumed the appearance of a small bladder or cyst, with a rounded aperture at one of its extremities.

2. This was a piece of a calcareous tumor removed from the body of a female, who had passed portions of the calculi from the uterus during life. It appeared to consist of a number of irregularly formed masses, of a white and somewhat chalky-looking substance, connected together by an animal matter, of a brown colour, and solid consistence, which had the appearance of inspissated mucus. Passing over the chemical processes resorted to, which indeed were similar to those employed in the preceding instance, we may state the muriatic solution was found to contain a large quantity of carbonate of lime, with a small quantity only of the phosphate, the proportion being nearly as ten to one; it also contained the sulphate of lime, in about the same proportion with the phosphate. The animal matter appeared to be identical with what Dr. Bostock formerly described under the title of albuminocerous matter. Altogether Dr. B. sums up:—"From the above experiments we may conclude, that the substance in question consisted of an earthy compound, composed principally of carbonate of lime, united to a small quantity of the phosphate and the sulphate of lime; it was imbedded in or connected with an animal matter, which appeared to be, for the most part, coagulated albumen, with minute quantities of the peculiar substance found in the serosity of the blood, and of what I have termed albumino-serous matter. From the aspect and general character of the body in question, I should conceive that there is no organic connexion between the earthy and the animal matter of which it consists.

3. This was from the uterus. It was an irregular mass, somewhat resembling

No. 1, but with less distinct concretions, the earthy and the animal matter being more intimately mixed together. Subjected to the same processes it was found to contain a considerable quantity of the phosphate of lime, with small quantities of the carbonate and the sulphate. It appeared therefore that the earthy matter of this substance was nearly similar to that of No. 1, and that it was, in like manner, attached to a portion of an animal matter, which resembled coagulated albumen.

4. From the arch of the aorta. It consisted of a white matter, imbedded in a hard, semi-transparent substance; when torn or divided, the white matter exhibited somewhat of a fibrous texture. It contained a minute quantity of albumen, a trace of gelatine, and other matters—a considerable quantity of phosphate of lime, a little of the carbonate, and a trace of the sulphate. "It may be considered as consisting essentially of the earth of bone imbedded in a membranous body; the earthy matter being in the form of filaments or thin plates, interspersed through the substance of the animal matter."

5 Calculi from the lungs. The specimen consisted of three small bodies, of which the largest weighed rather more than four grains, and the two smaller about half a grain each. They had all the same appearance, that of a white granular mass, of an irregular form, of considerable hardness, and an earthy fracture. They were made up of a large quantity of the phosphate of lime, a small quantity of the carbonate, and a trace of the sulphate.

6 and 7, were also from the lungs. Their composition was so similar to that of No. 5, as not to require special notice.

"The results," adds Dr. Bostock, "of the above analyses confirm the opinion that had been previously entertained respecting the chemical composition of these bodies; that they are, for the most part, composed of the phosphate of lime and animal matter, generally united to a small quantity of carbonate. Out of the eleven concretions which I have examined, the only exception is that which was taken from the uterus. This, both in its physical and chemical characters, differed very materially from the others, while it more nearly resembled the salivary concretion that was analyzed by M. Lassaigne."

8. This was a large pendulous bony tumor, connected by a short strong peduncle to the back part of the fundus uteri. It was obviously compounded of two substances; a dense and hard body, intermixed with layers, or deposited in the cells of a membranous substance. The exterior surface was irregularly tuberculated, while the interior exhibited a number of cavities of various forms and sizes. The earthy and the animal matter appeared to exist in very different proportions, in different parts of the calculus; Dr. Bostock took ten grains of what appeared to consist of an average proportion of the two, and subjected them to the same mode of analysis, as was employed in the former experiments. The result was, that the ten grains were composed of—

| | |
|---|---|
| Animal matter .... | 3·2 grains |
| Phosphate of lime.. | 6·45 |
| Carbonate of do.... | ·35 |
| | 10·00 |

9. This was from a case of Dr. Chownes, related by Dr. Lee in the Society's Transactions. Five grains were examined, and their composition, setting aside the details of the processes employed may be set down thus:—

| | |
|---|---|
| Jelly, mucus, &c. ...... | 2·1 |
| Membrane, &c......... | 1·1 |
| Phosphate of lime.. ... | 1·5 |
| Carbonate of do. ...... | 0·2 |
| Sulphate of do......... | 0·1 |
| | 5·0 |

10. A portion of a calcareous tumor of the uterus. It consisted of a number of tubercular or vermicular bodies, and forming an irregular concretion, and having in the interior cavities of various forms and sizes. The proportion of the calcareous salts was as follows:—

| | |
|---|---|
| Phosphate of lime ...... | 8·5 |
| Carbonate of do. ........ | 1·45 |
| Sulphate of do. ........ | 0·05 |
| | 10·00 |

Dr. Bostock concludes:—

"From these three analyses, taken in connexion with those in the body of the paper, we may conclude, that these earthy concretions, in whatever part of the body they may be deposited, are nearly similar in the nature of their constituents, although with some variation in the proportions; that in almost all instances, the phosphate of lime is the predominating substance, while, in certain cases, the carbonate would appear to be the principal ingredient."

This is a valuable chemical addition to the stock of pathological facts. We trust that chemistry will be more and more applied to the investigation of the nature of morbid structure.

### Suppression of the Urinary and Alvine Secretions for several Years.

Dr. Montesanto has related this almost incredible case in an Italian journal. The individual (Dom. Valetto) had sustained an injury on the loins nearly 18 years ago, followed by weakness of the lower extremities, and difficulty of discharging urine and fæces. He was committed to prison, in Padua, for some crime, and was there seized with inflammation of the medulla spinalis, succeeded by paralysis of the lower limbs. The patient vomited daily the ingesta which he had taken—and complete suppression of the renal and intestinal evacuation now took place.—After some time stercoraceous matters were vomited up, as well as partially digested substances. In this wretched state he continued eight years, viz, till 1828, when M. Montesanto took him in charge. The stercoraceous vomiting now became less frequent, and was sometimes absent for a considerable period of time. Inflammatory and congestive symptoms rendered bleeding very often necessary. The inexorable tribunal of justice, devoid of humanity, refused all applications to have this victim transferred from a loathsome cell to the wards of an hospital!! He lingered out a miserable existence of 17 years, and expired in February, 1835.

*Dissection.* Nothing particular in the brain, Vertebral canal normal. In the canal itself was found much sero-sanguinolent fluid. The medulla spinalis was apparently free from disease. The stomach was preternaturally large, and contained some yellow and fæcal matters; but its coats were unaffected. The gall-bladder was void of bile, but contained a number of biliary calculi. The small intestines presented several contractions, particularly in the vicinity of the cæcum. They contained the same kind of matters as the stomach. There was nothing remarkable in the colon above the sigmoid flexure. There the parietes became thickened and contracted, and the mucous membrane presented several ulcerations. There was fæcal matters throughout the whole of the alimentary canal—very fluid in the small, but dry in the large guts. The liver was normal. The bladder was contracted, and its coats thickened. It contained a few drops of thick urine. The kidneys were healthy — ureters flaccid.

The appearances on dissection are very far from accounting for the strange phenomena during life. We are not without suspicion that some deception was practised on our Italian friends. The darkness of a dungeon, and the hopes of mitigation of sentence, were circumstances favourable to such attempts. The discipline could not have been very rigorous, for we are told that he often indulged in large potations of brandy! But the statements respecting the *suppression* of urinary secretion, for so many years, contrary to the acknowledged condition of the urinary apparatus, as proved by dissection, and contrary to all experience, pathological and clinical, stamps the whole case as calculated to make the ignorant stare, and the credulous to wonder. We have just as much credence in this case, as we have in the spontaneous liquefaction of the blood of St. Januarius! Some of our contemporaries have been finely hoaxed by Dr. Montesanto!

---

INTENSE HEADACHE (IN PAROXYSMS) REMOVED BY TONICS AND LOCAL BLEEDING—(DR. BRIGHT).

This case is related in the first No. of Guy's Hospital Reports. The patient was J. King, aged 50, who was admitted in February, 1835, labouring under intense headache, somewhat periodical, of ten months' duration. The pain arose from the nape of the neck, extending to the front of the head, coming on in paroxysms, and attended by giddiness, and sometimes loss of consciousness. No external tenderness—no paralysis—pulse 84. The nitrate of silver was applied to the shaven scalp, and aperients were ordered. Five days afterwards he was not relieved, and, in the paroxysms, the pulse fell to 52, rising, as the pain abated, to 64. Senna was given, and cupping-glasses applied to the nape of the neck. The ferri subcarbonas was also prescribed, in 40-grain doses, every four hours. In four days more, the paroxysms were found to be more frequent than before. The dose of the carbonate was doubled. Senna draughts, with colchicum, were exhibited. In two or three days more, the cupping was repeated, and the arsenical solution was prescribed, with cascarilla. Afterwards the quinine was ordered, and taken for a fortnight, with a couple of weeks' freedom from pain. In this state, the patient was discharged; but whether or not he was permanently cured, the deponent saith not.

---

EXTIRPATION OF THE GREATEST PORTION OF THE RIGHT SUPERIOR MAXILLARY, AND THE ANTERIOR PART OF THE MALAR BONE, WITH A FIBRO-CARTILAGINOUS-LIKE TUMOR, WEIGHING TWENTY-ONE OZS. By RICHARD TUTHILL, M.D. Surg.

[Received through the Army Medical Board.]

James Samuels, aged 25, a black, in the parish of Irelanny, Jamaica, healthy-looking, with the exception of a large morbid growth, occupying the right side of the face.

Reports that, when a lad, he received a kick from a horse in the right cheek. When grown up, he for the first time felt a small lump, like a kernel, in the same spot. This lump was situated external to the centre of the antrum, and has gradually increased, from the centre to the circumference, to a most enormous size, rendering the man a most frightful and disgusting spectacle, and his existence an annoyance and burden to himself. He experiences an unceasing pain, shooting through the orbit, sometimes through the whole head and face, with a fetid discharge, and occasional hemorrhage to a great extent.

The surface of the tumor is smooth; the integument thin, and apparently much stretched. Numerous small veins run from within outwards, and from above downwards. The tumor is perfectly firm and unyielding. Its measurement, previously to being extirpated, was as follows—eight inches and a half, in an oblique manner, from the inner canthus of the eye, towards the external angle of the lower jaw; seven and a half inches from the septum narium towards the ear; and five and a half inches in a perpendicular direction, from the inferior lid to about the centre of the lower jaw. It preserves a well-defined edge, of tolerable thickness, occupying the anterior third of the malar bone, the superior maxillary and right nasal bones externally. The palate-bones are engaged in the disease. The alveolar process is turned upwards and outwards, and very much increased in thickness. The two incisors of the right upper jaw and the bicuspid are wanting, whereas all the teeth are complete in the opposite jaw. A large aperture, of long standing, is observed leading from the mouth into the antrum, through which he has been accustomed to keep a piece of linen plugged, to prevent the constant discharge of very offensive matter from the cavity, which is large enough to admit the middle finger to pass into it. Speech and deglutition are much interfered with. The tumor projects about four inches, in the centre, from the ordinary surface of the face.

January, 1830. Dr. Tisdale extracted the first molar tooth, which afforded an immediate vent to some very offensive matter.

*Operation, 22d March*—in presence of Messrs. Parkins, Dalrymple, and others. The common carotid was first encompassed by a ligature of reserve. An incision, five inches long, was then made, from near the inner canthus of the eye to near the commissure of the mouth. Another was made from the ramus of the jaw, parallel to the teeth, below the parotid duct, to join the former incision. The triangular flap was dissected backwards, and then the remaining integument and muscles were dissected towards the left face, as far as the septum narium. The superior maxillary and the anterior part of the malar bones presented an appearance resembling the shell of a crab, expanded over the anterior surface of the tumor. Hey's saw readily penetrated the shell, and the removal of the tumor appears to have been boldly and dexterously performed. The description of the different steps, it would be totally useless to describe, as it would be quite impossible to comprehend it. In this respect, our operator falls into the error of most other vivisectors. Suffice it to say, that the anterior third of the malar bone, exclusive of the orbital plate—the superior maxillary bone, excepting its orbital portion—the right nasal—the right palate—and a small portion of the left—a piece of the *left* superior maxillary, were removed, principally by the knife. It was then astonishing to contemplate the great structural alteration in the palate, nasal, and maxillary bones. They were softer than cartilage. The labial and orbital arteries were tied, and the facial vein. The ligature of reserve was removed from the carotid, and the edges of the wound were brought together by suture and adhesion.

The tumor was the size of a child's head of six or seven months' old, and was of cartilaginous consistence, without any vestige of a cyst. There was a thimble-like cavity in the situation of the antrum of Highmore, lined by a smooth membrane. The after-treatment was judicious and successful. This formidable operation does great credit to Dr. Tuthill.

---

### The Nakra, a peculiar Indian Disorder.

Mr. Twining, in his recent work on the diseases of Bengal, has given the description of a singular affection, confined to the natives of India, and denominated Nakra. It is a febrile disorder, which, though transient, and seldom fatal, is very severe and distressing. It seems to consist of a sudden and violent congestion of blood in the Schneiderian membrane, which extends to the mucous membrane lining the sinuses of the frontal, malar, maxillary, and æthmoid bones, with pyrexia, remarkable rather for the rapidity of the circulation than for augmented force of the heart and arteries. The invasion is sudden. and the sufferings of the patient great, from pain in the sinuses. Nakra means the nose-disease. The sensation of pain and distention within the nose is attended by extreme pain in the back of the neck—heat of the forehead—great weariness, and pain in the loins and joints. In a few hours, the pains are much augmented in the frontal and other sinuses, and in the nose; but without relief of the headach and pains in the loins. The eyes soon become red, with intolerance of light and prostration of strength. Thirst is distressing, and the patient takes to his bed. The pulse is rapid, but seldom full or hard. Mr. T. has known it rise, in a few hours, to 128, in an elderly Hindoo. The respiration is hurried, but not laborious; and much anxiety is expressed, especially in the standing posture. On examination, the Schneiderian membrane is found red and swollen. A burning heat comes over the wholebody, which continues some days. The ordinary duration of the complaint is from three to five days. It attacks both Hindoos and Mahomedans, of all constitutions and temperaments. Women suffer more rarely than men, and children under ten years not at all. Some people escape it entirely, and others have it once a fortnight for three or four months. Generally speaking, those who have had the nakra twice, are subject to annual attacks for many years afterwards. It does not recur at any particular period. Irregularities in living, and unnecessary exposure to the sun, are supposed to be the causes. They employ very little medicine for the complaint, and usually wait with patience till it is over. The natives of Bengal, however, are accustomed to draw blood from the nose, by means of sharp grass thrust up the nostrils, or by puncturing the Schneiderian membrane with an awl. This affords remarkable relief.

The nakra appears to us to bear more resemblance to the HAY-FEVER, than to any other complaint with which we are acquainted. Dr. B. Rush, however, mentions a curious complaint, known in some parts of America by the name of *pleurisy in the head.* This is probably analogous to the nakra.

---

### COMPARATIVE PATHOLOGY.

### PALSY IN THE HORSE.

The progress of medical education has received an immense impulse within these last few years. It has increased, is increasing, and must continue to increase, in an accelerated ratio. We are not of the alarmist school. We do not look with horror or with dread at this. It is a part of the great mouvement of society, and, whatever may be thought or said by individuals, it *must* go on. It is in vain for particular feelings or interests to struggle against such a tide as this.

The alterations in the quantum of what is taught to medical students, are equalled by the alterations which have been effected in its nature. Regard anatomy. How different is the mode of teaching it now, from what it was a few years ago. Yet still comparative anatomy is neglected, and does not form a necessary component part of the studies of the surgeon or physician. That it must do so ere long is obvious. Attendance on lectures on comparative anatomy must form a part of the system of education insisted on by the new university. It would be monstrous not to include it.

Mr. Youatt, in some lectures published in a monthly journal, the "Veterinarian," has made some very pertinent remarks on the value of comparative morbid anatomy. This is a subject which has hardly yet been broached; yet it is obviously one of high interest, if not of actual importance.

Mr. Youatt, from his situation as veterinary surgeon to the Zoological Society of London, must of course enjoy such opportunities of cultivating comparative pathology, as have fallen to the lot of none before him in this country. Those opportunities will not be neglected, nor could a fitter man be easily found, for what we must deem the responsible office that he fills. We say responsible, because Mr. Youatt probably has it in his power, by a careful and exact observation of the facts that daily present themselves to him, not only greatly to advance his own department of medicine, but human pathology too. We would, therefore warmly urge him to communicate, from time to time, to the profession the results of his researches, and we will take care to extend the knowledge of comparative pathology, as we endeavour to promote the acquaintance with the human, to the utmost of our power. At present, we have only time to notice a few facts connected with palsy in the horse. In our next, we will endeavour to introduce a more extended series of facts, which may appear to be instructive or curious.

Mr. Youatt remarks that general palsy is rare in the horse, while partial paralysis is not only frequent, but sometimes fatal. The following case of paralysis, depending on inflammation of the membranes and substance of the spinal cord, is interesting.

*Case.* "An old pupil and valued friend of mine, Mr. Chapman, who kindly conducted my business for me when I was ill, was sent for, in order to examine a horse. The symptoms were, heat of mouth, heaving at the flanks, a hard and quick pulse, and the exhibition of great pain. As he stood by his side and looked at him, he observed some slight spasms of the right shoulder, and extending along the side. He naturally concluded that it was a case of pleurisy, and he bled the animal and gave fever medicine.

On calling a few hours afterwards, he found the horse in still greater pain, and his left leg was becoming powerless. The horse could not bear upon it, and he shrunk at the slightest touch." "He soon after this fell, and beat himself dreadfully about. My young friend began to understand the case: he ordered the limb to be frequently fomented, he rubbed in a stimulating liniment all over it, and he gave a strong dose of physic. On the following day, he began to give opium and spirit of nitrous ether.

The horse had then been set on his legs with a great deal of difficulty; he stood continually shifting his position, so far as he could do it with his hind feet, and trying in vain to rest a little of his weight on the affected limb. At length the power of the other leg began to fail, and he again fell down, and, unless forcibly held down, beat his head about, and fought and scrambled with his hind feet. He survived until the fifth day after the attack.

On examining him after death, there was scarcely any thoracic or abdominal lesion, but there was spinal disease from the fifth to the seventh dorsal vertebra. The membranes of the chord were inflamed, the ligamentum denticulatum very much so; the inferior columns were pulpy, and the fluid in the central canal was black. The superior and lateral columns presented no lesion."

The same symptoms were exhibited by the horse, as are sometimes seen after injuries of the spine in man. When the cord and its membranes become much inflamed, the paralysis which previously existed sometimes seems to pass away, and horrible pains are felt in the affected limbs. The paralysis returns, and the patient sinks. This case has been well pointed out by Sir Charles Bell.

It is odd that hemiplegia, the most common form of palsy in men, is exceedingly uncommon in the horse. In other words, apoplectic effusions on the brain are rare in that animal. Mr. Youatt appears to attribute the circumstance to two causes—the absence of mental excitement, and the regulated diet of the equine patient. There are cases related of two stallions, who became paralytic after covering too many mares.

"Palsy in the horse proceeds from injury of the spinal chord, and that chord is more developed than in the human being. In man, the brain may perhaps be averaged at one thirty-fifth part of the weight of his body. In the intellectual dog, it can be rated at little more than the one hundred and sixtieth part. In the horse, it is one four-hundredth part only; and in the ox one eight hundred and sixtieth. Not only is the bulk of the brain smaller in the quadruped than in the human being, and therefore less likely to exercise so paramount an influence over the chord, but the spinal chord, and especially the inferior or motor surface of it, is, *compared with the bulk of the animal,* very much more developed in the quadruped than in the biped. The brain of the horse is smaller than that of man; the spinal chord is considerably larger. Compare the relative bulk of the brain and that of the chord in man and the horse: compare, also, the full rounded development of the motor columns on the inferior superficies of the horse with that of the same columns on the anterior face of the spinal marrow of the human being. The principle of intellect is developed in the one; the other is chiefly formed with reference to muscular strength and endurance.

The spinal chord is more exposed to injury, and injury which will affect not one side only, but the whole of the chord at that part. It is also an anatomical fact, that the decussating fibriculi which extend from one motor column to the other, and connect the two together in consentaneous action in the animal whom we often tax so severely, and whose utility to us consists principally in his muscular strength, are far larger in the horse than in man; and, by means of these connecting fibres, disease also is more likely to spread from side to side. It is on these accounts that while hemiplegia is the form which palsy oftenest assumes in the human being, paraplegia, or palsy equally affecting both sides, is the most frequent malady of the quadruped."

Palsy usually affects the hinder extremities, because, in consequence of their articulation to the pelvis, the strength of their muscles, and the exertions they are called on to perform, injury to the corresponding portion of the spine is most frequent.

Mr. Youatt informs us that in palsy dependent on lesion of the spine, the case is almost always inflammatory and requires active antiphlogistic treatment. Here is a sketch of palsy of this description.

"If you inquire into the history of the cases that come before you, you find that the horse had fallen, and had suddenly become paralytic—or palsy had supervened a few days after the accident—or he was seized in the midst of his work, and when his energies were too cruelly taxed—or he had been worked exceedingly hard a few days before, and there had been a heavy load—or the pace had been greater than usual—or, covered with perspiration, the horse had been left exposed to the cold and wet."

"Generally speaking, there are few precursory symptoms. On the previous day, the horse is apparently well; he is found on the following morning, or soon after some severe accident, dreadfully lame; in great pain; and shifting his weight from one limb to another. In some cases there may be previous fever, heaving, illness which can scarcely be referred to any particular part, or derived from any particular cause. Very shortly, however, the mischief can be traced to one leg; perhaps both are equally affected: the animal can scarcely walk; he walks on his fetlocks instead of his soles; he staggers at every motion; he hurries along to prevent himself from falling; at length he falls; he is raised with difficulty, or he never rises again.

The sensibility seems for awhile to be very much increased, but the feeling of the part, and the sensibility generally, gradually subside—they get below the usual standard—they cease altogether. There are none of those sudden suspensions of feeling and voluntary motion which are recorded of the human patient, because it is not so often an affair of the head, It is the result of spinal disease or injury—it originates in inflammation of the spine or its membranes, whatever be the cause of that inflammation; and it is ushered in by fever and excruciating pain. When the pain which accompanies the first attack has passed over, the animal, with the exception of his powerless limbs, appears for a while—a time of very uncertain duration—in the full possession of all his senses, and eats and drinks as usual."

On dissection of such cases, there is almost always inflammation of the cord or its membranes about the lumbar region.

Palsy in the ox and cow is not unfrequent after exposure to damp and cold. The cow is particularly exposed to palsy at the period of calving, or shortly afterwards.

Calves, if turned out too soon after weaning, are very subject to palsy.

In all these cases, we are told that there is usually inflammation of the spinal cord or its membranes. The great art in the treatment seems to be to get the beast into such a state that the butcher may make the most of him.

---

### New Remedy in Epilepsy.

We shall quote some extracts from a paper which we have met with in a recent American Jonrnal,* relative to a remedy little, if at all known, in this country: viz.

"*On the Crusta Genu Equini (Sweat or Knee Scab, Mock or encircled Hoof Knees, Hangers, Dew Claws, Night Eyes, or Horse Crust,) in Epilepsy.* By John P. Mettauer, M.D.

"The substance designated by the several appellations at the head of this article, is furnished by the horse; four oval secreting surfaces, situated on the inner aspects of the extremities, near the knees, are the parts of the animal from which it is obtained. The secretion is poured out so gradually, and in such small quantities at a time, as not to be observed in its fluid, or even semi-fluid states. The crust is of variable color, as well as density; its exterior is always of a lighter appearance, and harder than the interior, which is dark and soft; it is of a lamellated and fibrous texture, and when broken, resembles dark, soft horn; its odor is very penetrating, diffusible, and peculiar; it is deciduous, and separates gradually two or three times during the year; when prematurely or forcibly removed, the surface from which it is taken sometimes bleeds a little, inflames, and becomes tender and sore."

"As a *medicinal agent*, the crust has been long known in this part of the country. How it found its way into use as a remedy, is not certainly ascertained. It is conjectured that the coincidence of the horse being observed to bite the crust, and to pass worms from the bowels soon after, suggested it as such, and the conjecture is by no means improbable, when it is remembered that this article was first employed as a vermifuge with that animal. The fœtid odor of the crust, it would seem, might naturally have suggested the idea of its possessing remediate powers, and doubtless did indicate it as a nervine and antispasmodic, after it was supposed to possess vermifuge properties.

We have long known and employed this substance as an antispasmodic; but the merit of introducing it into regular practice, is due to Dr. Joseph Mettauer (the writer's father), who employed it in epilepsy so early as 1782 or 1783. During the last twenty-five years we have enjoyed many and satisfactory opportunities of using the crust as a remedy in epileptic convulsions.

In collecting the crust for medical purposes, it is necessary to attend carefully to its loosening tendency from time to time, or it may fall off and be lost. It may be made to separate a little sooner by gently soliciting, and occasionally by firm compression with a bandage. This should be suffered to remain on after the period of desquamation is near at hand, to prevent the accidental loss of the crust. After it is obtained, it should always be dried a short time in the shade, and then it may be kept for use in a close jar, to prevent, as far as possible, the escape of its volatile properties."

"Two forms for administration are used—the powder and tincture. When the powder is to be used, it should always be freshly prepared, either by pounding and rubbing the dry crust in a mortar, or by grating it with a common nutmeg-grater; this last process will be found (generally) most convenient, as it enables the practitioner to reduce it, at once, to a very fine and equable powder, even if the crust is imperfectly dried.

The tincture is prepared by simply digesting the broken or powdered crust in diluted alcohol, or common brandy, exposed to a gentle heat for eight or ten days, in the proportions of one part of the former to four of the latter.

The doses of the powder vary from two to twenty grains; it may be given diffused in any liquid which the patient fancies. With young patients it is safest to begin with the minimum, and increase very gradually to the maximum doses. Should the disease yield before the largest doses are reached, no further augmentation need be made. When the tincture is employed, from ʒss. to ℥iss. are its extreme doses. Diluted with water and sugared, it may be given

* The United States Medical and Surgical Journal, Oct. 1835.

with very little difficulty to the youngest subjects, as it is tasteless, and in a great measure inodorous. In this form, also, the doses should be very gradually increased, to prevent, as far as possible, the danger of exciting the system too much, which might result from the menstruum, should the doses be suddenly augmented."

"With young subjects from six to eight years of age, two grains will, in a majority of cases, constitute the commencing dose. We have never used it with patients younger than six years, or older than thirty. Older patients, say from eight to twelve, or fifteen years of age, will bear four or five grains, or even larger doses in the commencement, and with such it may be more suddenly increased to the maximum doses, without gastric disturbances. The remedy rarely offends the stomach when the foregoing precautions are properly attended to; on the contrary, it seems rather to compose and tranquillize this organ. Three doses, in a majority of cases, are as many as will be required in the twenty-four hours. Should cases occur marked by convulsions of unusual violence, with frequent paroxysms, it may be given oftener. From many trials with this article, it has not been perceived that there is much diversity of effect when employed in large or medium doses with young subjects."

The author remarks that, in obstinate cases, the crust should be continued for more than a year before it is discarded. He does not prohibit the use of suitable remedies as auxiliaries, during its administration.

---

### Ovarian Dropsy accidentally cured. Dr. Addison.

[Guy's Hospital Reports.]

A female, aged 44, the mother of one child, was received into Guy's Hospital on the 19th March, 1834. She had had ovarian dropsy for some years; but, nine days previous to entering hospital, she fell, and a pair of steps, on which she had been standing, fell across her abdomen. She immediately suffered excruciating pain—became sick and faint—and soon perceived that the fluid accumulation which had been circumscribed, was now diffused over the abdomen, rising to the diaphragm and obstructing respiration. She had then, no doubt, an attack of peritoneal inflammation, for which she was treated—but afterwards entered the hospital. Her abdomen was now distended with fluid, and very painful—pulse 98—urine copious—and she had passed blood by stool—she was bled, fomented, and took calomel and opium, under which treatment she improved. Her mouth became sore on the 22d, after which the fluid rapidly decreased. On the 5th April no fluctuation was perceptible. Still the remains of the cyst could be felt stretching across from one iliac fossa to the other. She had afterwards an attack of phlegmasia dolens, which was soon cured. She is now living as a servant in Cheapside, and can yet distinguish a small tumor in the left iliac region. She has had no return of the dropsical enlargement.

We fear that it would not be quite safe to imitate this accidental mode of curing ovarian dropsy.

---

### Case of Loss of the Uterus and Appendages soon after Delivery. By John Charles Cook.

Mr. Cooke has published this case in the form of a pamphlet. The case itself is of too interesting a complexion to pass unrecorded in the pages of this Journal.

At 4 a. m. of the 22d of May, 1835, a midwife of Coventry, was sent for to a woman in labour. On her arrival, she found the woman, who had already been in labour 48 hours, upon her knees, and insisting upon being delivered in that position, as being the custom of her country (Ireland).

With some difficulty she induced her to lie upon the bed, in order, by an examination of the parts, to ascertain how far the labour had advanced. Upon this being done, the Os Tincæ was found dilated to about the size of half-a-crown; the child's head presenting as usual. Every thing went on well, and the woman was delivered at seven o'clock the same evening of a living child. The placenta followed whole in a quarter of an hour, being expelled by a pain.

No hæmorrhage whatever ensued at the time, although a considerable quantity of blood was lost during the night. The after-pains were trifling, and when the midwife visited her the next morning, she appeared in a very satisfactory state. So well, indeed, did she feel, that notwithstanding the strict injunctions of the midwife to the contrary, she partook plentifully of animal food, immediately after her departure. At 4 A.M. of the 24th, the midwife was again hastily summoned. She ascertained the following particulars :—that the woman had risen during the night, and had gone into an adjoining room to make water; that whilst there she had, by her screams alarmed her husband, who called in some of the neighbours, and upon entering the apartment, they found the woman seated on a stool, before the fire, with a vessel of warm water in front of her, and a large substance, which they compared to a child's head and neck, lying between her thighs, supported by her hands. They then sent for the midwife, believing it to be a case of twins, and being greatly alarmed at the quantity of blood she had lost, the hæmorrhage having been profuse. She was now lying on the bed, pale from the loss of blood, which had been excessive. On examination, the midwife discovered a hard substance, lying on the bed, loosely connected to the vagina by a shred of membrane only. This substance she immediately recognized as the Uterus, and lifting it gently, removed it without difficulty or effort, and placed it in a wooden bowl. The hæmorrhage then ceased, and the midwife, after a short delay, left the house, taking the uterus to our author's father. He found that it really was an uterus inverted. The only part absent was the left ovary. Mr. Cooke, senior, visited the woman at 11 A.M. and found her completely exhausted from the hæmorrhage, (which by this time had ceased); she was extremely restless and agitated, constantly throwing her arms about; pulse scarcely perceptible. Upon enquiry he found she had passed some urine shortly after the loss of the uterus, as also between 9 and 10 the following morning (Saturday). He was also informed that her bowels had not been relieved for a period of at least 9 days before delivery, except a mass of hardened fæcal matter which was discharged, with the last pain in the labour. The woman did not much complain of any sensation like bearing down, nor of any substance lying in the vagina. She did not appear to suffer much from pain: neither was there much distention of the Abdomen, nor was there then or at any time during the progress of the case any thing amounting to more than a slight degree of tenderness, which, however, was hardly noticed except upon pressure. As there was no protrusion of the pelvic or abdominal viscera, and as there were no urgent symptoms, Mr. C. determined not to incur any risk by instituting an examination per vaginam. He merely enjoined quietude and the horizontal posture, with a light farinaceous diet.

In the afternoon the pulse rose to 140. The patient passed her urine freely during the night, and next day presented no unfavourable symptoms. In short, no material symptoms ensued, and she perfectly recovered without any surgical, and scarcely any medical treatment.

Previous to her confinement, milk was secreted in considerable quantity, but immediately after the loss of the Uterus this secretion, together with that of the Lochia was arrested. Notwithstanding this, and in spite of the remonstrances which were made to her on the subject, she persisted in applying the child to the breast, which induced considerable pain and hardness of the right mamma, attended with a good deal of febrile excitement. These symptoms subsided upon the exciting cause being removed. When her health had been in some degree re-established, she again gave the child the breast, and persevered in doing so during several weeks, until finding that she had no milk she finally desisted. The child has since died of diarrhœa.

"Owing to her constant and obstinate refusal to permit any examination per vaginam to be made, after her recovery, I am not able to give any description of the state of the parts at the present moment: the most probable supposition is that the vagina forms a cul-de-sac in consequence of the adhesion of its opposing surfaces. In this manner we may easily account for the viscera remaining in situ after her return to her ordinary active habits. It will be recollected that considerations of this nature, induced my father to refrain from instituting an examination when first called in. This idea is further strengthened by the fact, that sexual intercourse has repeatedly been had with her husband, no mechanical obstruction existing.

In connexion with the loss of one ovary, it is interesting to be able to state, that, although no impediment to coitus exists, yet the usual feelings and desires are entirely wanting. This might have been anticipated had both ovaria been removed, but from the fact of one of these bodies still remaining in situ, some degree of doubt might have been entertained.

Of course, after the loss of the secreting organ, the menstrual discharge has never re-appeared; even the small quantity occasionally furnished by the vagina has not been observed."

This is certainly a curious case, and it appears by no means clear, why the uterus should thus be torn away, as there seems to have been no violence employed—or why, the uterus being removed, no descent of the viscera should occur. In the absence of any certain information on these points, speculation would be idle. The most interesting physiological consideration is the complete cessation of milk and of the lochia. A striking instance—the disappearance of the milk—of the mysterious connexion between even distant organs, associated in their functions in the animal economy.

The following is the description of the uterus. It is aided in the pamphlet by a lithographic sketch.

"In size it nearly equalled an ordinary child's head at term. No laceration of any kind was visible except a slight rent in the posterior lip of the Os Uteri, Fig. 1, C. The attachment of the Placenta was distinctly marked, being of a deeper brown red, than the remainder of the mass: its site was also rendered further evident, by a quantity of flocculent matter still adherent to the walls of the uterine cavity. It was inserted over, and concealed the opening of, the left Fallopian tube. This orifice was easily discovered by detaching with the nail a small portion of the loose flocculent substance already mentioned.

The vessels were large and tortuous, and their patent state sufficiently accounted for the great hæmorrhage which had occurred, both after the removal of the Uterus and during the night succeeding her delivery.

Upon making a section along its anterior aspect, the broad ligaments, with both Fallopian tubes, and the right ovary were discovered. The fimbriated extremities of the tubes were particularly clear and distinct, the left ovary appeared to have been detached, and to have remained in situ."

The case we repeat is interesting physiologically, as well as to the accoucheur, and Mr. Cooke has done right in publishing the particulars.

---

CASE OF NOTENCEPHALE. By Dr. HILDRETH, of Boston.

Dr. Hildreth has published this case in a pamphlet, which has been transmitted to us. We shall abstract only the main particulars.

The monstrosity was the third birth of a married lady, aged 35. There was no apparent reason for the occurence of the malformation. Labour took place about the eighth month. The fœtus was born within the membranes, so that respiration was impossible. But, for fifteen or twenty minutes after birth, the motions of the arms within the membranes were distinguished by the mother.

The fœtus, a female, very fat and perfectly formed, except in the parts to be described, measured thirteen inches in length, and weighed three pounds one ounce. It had no neck; its face looked upward, slightly inclining to the left.

The vault or arch of the cranium fell directly backwards and downward from the brows; its cavity was very small, containing less than an ounce of the brain; opening by a large occipital hole of a flattened oval figure, capable of admitting the points of three fingers, one side of the occipital hole being formed by the spinal column, which was open throughout its whole length. The bones composing the arch of the cranium were completely ossified, and covered with well-grown hair, which descended as low as the base of the scapula, each side of the cerebral tumor.

The mass upon the back was at once recognized as the brain, covered with its appropriate membranes, through which the convolutions were very distinctly seen, large, and imperfectly developed. Beneath the membranes, a small quantity of dark-coloured serum was diffused, lying mostly in the sulci of the convolutions, giving them a deeper shade than natural; the membranes also being of a deeper color than natural, the mass presented a purple hue, which was heightened by considerable quantities of coagula, interspersed through the substance of the brain.

The brain was invested externally by two thin, firm membranes. It was divided into two hemispheres by a falx, running obliquely down the back, from the right temple to the left sacro-iliac synchondrosis, parallel to a lateral curvature of the spine.

The skin, from the sacrum to the occiput, was split, or divided and turned aside, so that the base of the brain reposed upon the true bodies of the vertebræ, the spinous processes being cleft vertically and turned to the right and left. The investing membranes did not perfectly envelope the brain, that is, they were not continued under the organ, but terminated at the edge of the true skin.

At the circumference of the brain, where the membranes came in contact with the true skin and hairy scalp, there was the most perfect cicatrization of the two textures.

The substance of the brain was soft —distinct lobes were not made out—nor could the convolutions be separated from each other. Considerable quantities of coagula were diffused in the substance of the brain.

The brain below the occipital hole, which reposed upon the vertebræ, sent off no nervous filaments, and had no nervous connection with the spinal nerves below, so that these spinal nerves were without cerebral influence.

The vertebral canal was open posteriorly, save in the sacrum, the spinous processes being vertically split in the middle, and turned to the right and left. In the sacrum was an attempt to form a canal, which was filled with a mass of reddish grey nerves, each nerve being invested with a tough membrane of great strength. There were in these nerves reddish grains, appearing like adipocire. They were also deposited between the nerves.

" The posterior faces of the vertebræ, as has been remarked, were covered by well formed dura mater, which was continued up the vertebral column into the cavity of the cranium, which it lined throughout. A very small and thin membranous bundle of nervous fibres was seen passing down over five or six of the processes of the left side; but, other than this, no trace of spinal cord was seen. A few nerves were seen going through the base of the cranium; one bundle appearing like the par vagum, passing the foramen lacerum. The petrous portions of the ossa temporalia in the base of the skull, were moderately large; and the internal ear was most probably perfect, with its appropriate bones, canals, membranes and nerves; the foramen auditorium internum of the right pars petrosa was large and well developed for the admission of the seventh pair of nerves—the left pars petrosa smaller and without any apparent foramen. The membrana tympani of both ears was perfect, well preserved, and is beautifully shown in the skeleton.

The optic nerves were large and well developed, the organs of vision being quite perfect.

The spinal nerves were large and well formed; when traced from the abdominal side of the spine through the intervertebral foramina, they were found to retain their full size, and pass directly into the dura mater of the spine, where they abruptly terminated, being firmly united with this membrane. These nerves were of a very tough, firm texture; the dura mater of the spine being not a hollow tube as usual, but a thick, firm, solid membrane. The nerves of the trunk, the upper and the lower extremities, were all perfect and properly distributed."

The thoracic and abdominal organs were well formed, save that the two kidneys were consolidated into one, with three ureters. There was only one umbilical artery.

We need not dwell on the peculiarities of the skeleton.

The principal point of interest connected with the case, is the development of the spinal nerves, independently of brain or spinal cord—for they had no connexion with the former, and the latter was not present—another proof, if proof were wanting, of their comparative independence.

---

### Remarks on the Phosphorescence of Organized Bodies. By F. Tiedemann.

We have not yet been enabled to notice the first volume of a translation of the Comparative Physiology of Frederick Tiedemann, which has been given to the English public by Drs. James, Manby, Gully, and J. Hunter Lane. In our next number we shall probably direct more particular attention to this most valuable work. In the present instance we merely intend extracting a few remarks on a curious subject—the phosphorescence of organized bodies.

Organized bodies may be luminous either in the state of life, or when deprived of it.

Lifeless organic substances may shine by isolation, as seeds, starch, feathers, horn, &c.

Or, by the action of heat, as wax, oils, wood, &c.

Or, by friction, as the resins.

Or, during combustion.

Or, when electricity is excited.

During putrefaction, both vegetable and animal substances are luminous. In dead vegetables, an evolution of light is most frequently seen in the wood, particularly that of the root, but also of the trunk and branches, when it is decomposed by a moderate heat, moisture, and without full exposure to the air. According to Dessaignes and Heinrick's experiments, the phosphorescence only occurs at a medium temperature. It disappears in frosts and great heat. Hot water puts a stop to it, as also desiccation. The evolution of heat takes place in atmospheric air. Tiedemann is led to think that during the decomposition of wood an eminently combustible organic combination of carbon, hydrogen, and oxygen is produced, which, like phosphorus, burns and gives out light at the ordinary temperature. Possibly phosphorus itself is an agent in it, since the researches of Berthier have shown the existence of phosphate of lime in the ashes of very many woods.

Phosphorescence is much more common in dead animals than in vegetables. Most people are familiar with its occurrence in fishes. For the most part it begins a day or two after death, when the body is exposed to moisture in atmospheric air or oxygen gas, at a temperature from 12° to 18°.

"During phosphorescence in the air, a clear, liquid, mucilaginous matter is perceived on the surface of fishes, which becomes a little turbid, consistent and luminous. This phosphorescent substance may be abstracted by washing, being by this means made to combine with the water, which is thereby rendered luminous. If fishes are put into glass vessels with water, a glittering circle soon appears at the surface of the liquid; if the water is shaken, it becomes luminous. The light disappears in water that has been boiled and is freed from the contact of air, but reappears as soon as the air reaches it. Phosphorescence ceases immediately on the commencement of fetid putrefaction. From all these facts it follows that the phosphorescence of dead animals is preceded by a decomposition effected by the influence of heat and air, whereby a luminous fluid is produced and given out. Probably it contains phosphorus, which is disengaged from the organic combination, and is consumed by a slow combustion."

Living bodies are phosphorescent; both plants and animals displaying this peculiar property.

The flowers of several plants, as the nasturtium, Indian pink, &c. have been observed to disengage light, like sparks, in serene and warm summer evenings. Yet the accuracy of the observations has been doubted, and the causes of the phosphorescence, if it really takes place, are buried in obscurity. Pultney and Volta regarded it as an electric phenomenon. But Tiedemann thinks that more probably it is owing to the emanation of a combustible matter, perhaps a volatile oil, which enters into a kind of combustion under the influence of the air. The *Dictamnus albus* is said to spread around it, during the warm summer evenings, an atmosphere that takes fire on the approach of a candle, and gives a brilliant blue flame.

Many living animals, especially aquatic, exhibit luminous phenomena. Most of the sea-zoophytes, many crustacea, several mollusca, and even some fishes, evolve light. To them is supposed to be owing the phosphorescence of the sea. Yet if animalculæ produce this appearance, how is it we find it in the sea and not in rivers? In the fresh water of the latter there are infusoria enough, and yet we must go to the salt-water for the luminousness. An immense variety of facts and references are produced by M. Tiedemann in reference to this subject. He shews satisfactorily enough the phosphorescence of the sea-infusoria, the medusæ, the sea-worms, mollusca, crustacea, and some fish.

Many insects living in the air are luminous. This is the case with many worms and beetles, but especially with the female of the lampyrus noctiluca.

" It is of a light bluish or greenish colour, and seems to envelop the whole of the insect. The male fly has a soft and delicate bluish light. It requires a temperature of about 55.7° for its appearance. The light of the female glow-worm is of a light topaz colour, with rather a tinge of green. The hour upon a watch may be distinguished by it. The light of the male is of the same colour. The fire-fly produces two degrees of light, the one fainter than that of the glow-worm, but without intermission. The second is a vivid white light, intermitting instantaneously, like vivid sparks of fire suddenly extinguished. Its power of illumination exceeds that of the glow-worm and all other animal light. The intermitting light gives the appearance of a membranous veil being removed from the surface of the organ, and suddenly drawn over it. The glow-worm requires a mean temperature of 50.7° for its appearance."—*Translators.*

The following resumé of the numerous observations on this curious subject is interesting, and must close this notice.

" Phosphorescence ordinarily commences at the close of twilight, when the shining parts appear like some points, which gradually increase. If animals are confined in a dark place before sunset, they begin to shine a long time before twilight. If they be exposed to the light whilst shining, their brilliancy is sensibly diminished, but always returns in darkness. The light is extinguished at the dawn of day, except in two points of the last ring, which continue to shed a feeble light, as was observed by Razumowsky and Macaire. Macartney remarks, that insects do not shine in the evening, if they have been withdrawn from the sun's rays during the day. Macaire made the same observation, at least on the first day of the experiment. On the other hand, Todd and Murray found that all the species, although kept during the day in dark places, did shine less in the evening, and even much sooner than when they had been exposed to the day light.

Carradori, Brugnatelli, Macartney, Treviranus, and others, observed that the emission of light is at the will of animals. Mueller and Murray think that phosphorescence is voluntary only, inasmuch as animals can retire and conceal the luminous organs behind the opaque parts. Treviranus explains the phenomenon by the faculty which insects have of accelerating or retarding respiration, and by the influence which he asserts the air exerts over the intensity of the light. Macaire thinks the power of the will on the production of light cannot be denied, because any noise or a blow on the animal sometimes causes it to cease shining, and the light then disappears, except in the two points of the last ring. He attributes the cause of the phenomenon to the influence of the nerves. It is moreover certain that the light is augmented by the movements of the body."

The phosphorescence of living animals is dependent on the temperature of the air, occurring only, under ordinary circumstances, when that is above 12°.

The following are the sentiments of Tiedemann on the causes and the objects of this phosphorescence.

" Weighing well all the circumstances, phosphorescence would seem to depend on a matter, the product of the changes of composition accompanying life, and, to all appearance, secreted from the mass of humours by particular organs. This liquid probably contains phosphorus, or an analogous combustible substance, which combines with the oxygen of the air, or of aërated water, at a medium temperature, and thus produces the disengagement of light. The preparation and secretion of this substance are acts of life, which change, augment, or decrease by the influence of external stimulants, whose action on the animals modifies their manifestations of life. But the phosphorescence itself depends on the composition of the secreted matter, and cannot be regarded as a vital act, because, on certain occasions, it continues for whole days, even after the death of the animal. All that can be said on the utility of the light in the economy of shining insects, is that probably the preparation and secretion of the luminous matter is important to the preservation of life in these animals. Neither do we deny that this light may aid the sexes in finding each other at the epoch of copulation; at least it is observed that the males are attracted by the brilliant objects. Perhaps it may serve to protect them from the aggressions of enemies."

We shall return to this volume, containing, as it does, a mine of curious and valuable facts. In the mean time, we recommend it in the strongest terms to every professional man, young or old.

---

### CASES ILLUSTRATIVE OF THE INJURIOUS EFFECTS OF ADMINISTERING STIMULATING REMEDIES DURING THE INFLAMMATORY STAGE OF GONORRHŒA. By HENRY JAMES JOHNSON.

In the 38th and 39th numbers of this Journal I published some remarks on what appeared to be the most judicious mode of treating gonorrhœa. The principal aim of those observations was to deprecate the indiscriminate employment of stimulating remedies, during the inflammatory stage of the complaint. Since that period many opportunities have occurred to me, of witnessing the mischiefs that ensue from the empirical exhibition of cubebs and copaiba, and the rash or unguarded use of injections. I have no doubt that, in the great majority of cases, stricture of the urethra is the fault of the surgeon—the consequence of his disregard of antiphlogistic remedies. Whilst the urethra is in a state of inflammation, it is additionally irritated by the operation of the turpentines, or balsams, or the application of injections, or even the introduction of bougies. Those principles which regulate our treatment of the inflammation of other mucous membranes, are too frequently forgotten or disregarded in the case of the urethra, and surgeon and patient would seem to have made up their minds to the belief, that inflammation of the bladder, the penis, or the testicle, is a thing that may occur, as a matter of course—a part and parcel of the specific malady.

It would be going too far to assert that, under appropriate treatment, such inflammatory complications should *never* occur; but certainly they should *very seldom* occur. I am confident that their frequency will generally be in the ratio of the stimulating nature of the treatment. The opponents of antiphlogistic measures in the inflammatory stage of gonorrhœa, rest their objections on the tendency of those measures to occasion gleet. In the last two years, I have had the management of a considerable number of gonorrhœal cases. Of the patients whom I have treated two only have had gleet. In one, the discharge lasted upwards of a year, resisted every remedy I could imagine, and spontaneously disappeared on a visit to the country. In the other, the discharge existed for about two months, and was cured by blistering the inferior surface of the penis, and using an injection of the acetate of lead. I have not had, in the last two years, a single instance of hernia humoralis, or of any other inflammatory complication, with the exception of one case of inflammation of the absorbents of the penis. I certainly attribute this not to mere good fortune, but to the circumspection with which I make use of stimulating remedies.

My object at present is not to open up the question of the treatment of gonorrhœa, but simply to relate some examples of the effects of injudicious stimulation. If they succeed in awakening the attention of surgeons to the subject, and in making the less experienced pause in their employment of the measures that I deprecate, that will be sufficient.

### I. Inflammation of the Absorbents of the Penis.

*Case* 1. I was requested to see a gentleman who had lately arrived from Ireland, where he had contracted this, his first, gonorrhœa. Whilst travelling he had done little for it. On his arrival in London, about a week after its first appearance, he applied to a medical man. At that time there was much pain in micturition, and the discharge was yellow and abundant. His medical attendant prescribed cubebs. Next day the discharge was diminished, but there was much scalding, and the penis appeared rather swollen. The cubebs was continued, with purgative medicines. The discharge decreased, but the swelling of the penis augmented.

Three days afterwards, the patient came under my care. The penis was then considerably swollen—the integument was rather red, and two red lines extended along the dorsum of the penis to the pubes. On examination I found two hardened cords, almost as large as the thin end of a common clay-pipe, stretching up the dorsum of the penis, beneath the reddened lines. They commenced at the loose prepuce, and proceeded to the groin, where one of them joined an absorbent gland, enlarged to the size of a filbert. These cords were tender on pressure. The glans penis was rather swollen, the discharge yellow but scanty, the pain in micturition considerable.

Leeches, evaporating lotions, diluents, confinement to the sofa, and the lowest diet, were prescribed, in addition to calomel and ipecacuanha at night, and senna, with colchicum each morning. A week or ten days elapsed, before the swelling of the penis had disappeared, or the more decided inflammatory symptoms had vanished. At the expiration of that time there was still some scalding, and thickened cords were felt. Purgatives with demulcents were persevered in, and the mercurial ointment with camphor was applied to the penis. In about a week more discharge only remained. An injection of the acetate of lead and decoction of poppies removed this in the course of a week, and it never afterwards returned. I afterwards circumcised this gentleman, at his own request. He laid the whole blame upon his prepuce, and was determined to be quit of it.

I mentioned that a case of inflammation of the absorbents of the penis had occurred, although antiphlogistic treatment was employed in the first instance. The following is the case in question.

In June, 1834, I attended a gentleman, about twenty-two years of age, for his first gonorrhœa. The symptoms were decidedly inflammatory at the commencement, and it was necessary to have him cupped upon the perineum, in addition to other measures of depletion. The pain in micturition ceased in about a fortnight, and, at the end of the third week, copaiba was taken, and an injection of the acetate of lead employed. In a few days the discharge disappeared, and the gentleman appeared to be well. But, after lying down on the grass, he was attacked with what was thought to be rheumatic fever. This occurred at his own place, some little distance from town, and the gentleman was under the management of a physician. At the end of three months he recovered—a permanent stiffness of the right elbow remaining. I am inclined to suspect that this was probably "gonorrhœal rheumatism," rather than simply rheumatic fever.

In June, 1835, this gentleman again became my patient, on account of a second gonorrhœa. It may be mentioned that this was contracted from the same female as the first had been. He came to me immediately after the first appearance of discharge. There was much redness of the glans—copious thin and yellow discharge—much pain in micturition—and a feeling (probably nervous) of aching in the joints. Antiphlogistic remedies were prescribed. The discharge and scalding increased rather than diminished—painful erections were experienced—and the prepuce became red and swollen. This was at the end of the first week. In a day or two a hard cord could be felt, extending from the prepuce to the pubes, and terminating there in an enlarged absorbent gland. Leeches to the penis and a perseverance in antiphlogistic treatment removed these symptoms, in about three weeks from the commencement of the gonorrhœa, and cubebs, with injections of the acetate of lead and sulphate of zinc, effected a complete cure in ten days or a fortnight more.

Inflammation of the absorbents of the penis may be regarded as not an uncommon consequence of inflammatory gonorrhœa. It generally commences with slight redness and puffy swelling, from serous infiltration, of the prepuce. Then a red line is generally, but not always, seen on the dorsum of the penis; and, whether this exists or not, a hard cord is always felt. It usually proceeds to an enlarged and inflamed absorbent gland on the pubes, or in the groin. Occasionally, a circumscribed enlargement occurs on the dorsum of the penis, immediately behind the corona glandis. In one case, I saw this terminate in suppuration. This is consistent with what we observe in cases of inflammation of the absorbents in other parts of the body. The inflammation here and there extends from the lymphatic to the contiguous cellular tissue, and abscess in the latter is the consequence.

The treatment of inflammation of the absorbents of the penis is too obvious to require much notice upon my part. Leeches—evaporating lotions with the acetate of lead—calomel—purgatives—diluents—and rest on the sofa or in bed—must be employed with more or less vigour, in proportion to the quantum of inflammation in the individual case.

### II. ABSCESS IN THE CELLULAR TISSUE OF THE PERINEUM.

As the corpus spongiosum urethræ traverses a region—the perineum—abounding in cellular tissue, it cannot be a matter of surprise that inflammation should occasionally spread into that tissue, and occasion suppuration in it. This is sometimes productive of serious, almost always of troublesome consequences. The superficial fascia of the perineum, being closely connected with the fascia lata, at the tuberosity and ramus of the ischium, is apt to confine what matter may form, and to bottle it up in the perineal space. Between the superficial fascia which constitutes its floor, and the levator ani, with *its* fascia, which forms its shelving roof, is a mass of loose and adipose cellular tissue, offering no resistance to the deposition of pus, readily killed when that is deposited, and reposing behind upon the rectum, and in front on the urethra.

The cellular tissue, although it transmits the vessels of the other tissues, is not itself very vascular. The free communications between its cells permit the rapid accumulation of fluids, and these, when accumulated, speedily strangulate the filamentous structure of the cells themselves, cutting off their small supply of blood, and occasioning their partial or general sloughing. The series of changes from the simple effusion of serum and heightened vascularity of the cellular tissue, up to engorgement with lymph and pus, and consequent destruction, may be seen by incising a limb, in which subcutaneous diffuse inflammation exists.

Inflammation, then, of the cellular tissue of the perineum occasionally follows acute gonorrhœa, and may terminate in either of the following ways:—

1. It may form an abscess, pointing in the perineum, and communicating neither with the urethra nor the rectum.

2. The abscess may communicate with the urethra.

3. The abscess may burrow deeply, communicating with the urethra or rectum, or both, and even terminating fatally.

I need not detail any cases of simple perineal abscess, consequent on gonorrhœa. Most surgeons must have seen examples of it, and it requires no particular method of treatment. The surgeon, however, recollecting the disposition of the cellular tissue in the perineum, should leech freely to prevent suppuration; and, if that has occurred, should use the lancet early and not be afraid to plunge it deep. An early, a free, and a deep opening will frequently prevent much mischief.

The following is a case of abscess communicating with the urethra.

*Case.* When house-surgeon to St. George's Hospital, I received a young man affected with complete retention of urine. He had been labouring under gonorrhœa, attended with much ardor urinæ and chordee. For these symptoms he took cubebs. The discharge diminished rapidly, but retention of urine came on. Ineffectual attempts were made by the surgeon who attended him to introduce a catheter. At the time of his admission he could pass no water—had great spasm and pain at the neck of the bladder—and some tenderness on pressure in the perineum. I passed a small silver catheter into the bladder, but pus gushed out before the urine. As it seemed evident that an abscess existed in the perineum, which had probably been opened by the instrument, it was desirable at once to obtain a free external opening for the matter, in order to obviate the risk of extravasation of urine. I therefore cut down on the perineum, in the direction of the tenderness ; and, when the lancet was already up to its shoulder, out gushed nearly a tea-cupful of matter. The case terminated, as may be anticipated, in fistula perinei, which was not cured when the patient left the hospital.

The following is an instance of the fatal results that may follow perineal abscess.

*Case.* A groom, with gonorrhœa, was taking copaiba, when he rode a great deal on horseback. What he called chordee succeeded, and then he was unable to evacuate his bladder properly. Some few days after this I saw him. He was in a typhoid state. Urine dribbled from the bladder, which was greatly distended. There was a certain degree of fulness in the perineum. He was unable to answer questions rationally. I introduced a catheter with a great deal of difficulty, being obliged to give it a more abrupt curve than usual. An immense quantity of urine was thus drawn off. Supposing there was a putrid abscess, I cut with a scalpel deeply in the perineum, and let out much highly offensive matter. But the patient did not rally, and in two days afterwards he died.

On examination after death, there was found to be a very extensive sloughy abscess, stretching into the pelvis. All the contiguous parts were in a confused and sloughy state. The mucous membrane of the urethra was entire, although the muscles and cellular tissue surrounding the membranous part were involved in the suppurative and sloughing process.

In a young man received into St. George's Hospital, with nearly similar symptoms, the posterior wall of the urethra sloughed, as did the anterior wall of the rectum. Of course the patient died.

If the surgeon suspects the existence of abscess in the perineum, his great object must evidently be, to procure an external opening for the matter, and to prevent its communication with the canal of the urethra. For this reason, a bold and early incision should be made in the perineum, and the introduction of the catheter should, if possible, be avoided. In the first case which I related, there can be little question that the catheter opened the abscess, and, by establishing its communication with the urethra, occasioned perineal fistula.

### III. Inflammation of the Prostate and Neck of the Bladder.

The symptoms of inflammation of the bladder are familiar. Yet the earlier indications of inflammation of prostate and neck of the bladder would seem to be frequently neglected, and sometimes misunderstood.

The patient usually perceives that the discharge is lessened—perhaps that it has disappeared. At the same time, he experiences a sense of fulness, if not actually of spasm, deep in the perineum. He is obliged to make water more frequently than usual—he passes smaller and smaller quantities when he attempts to void it—the effort to do so is attended with spasm, or arrested by it—and, at length, the retention of urine is complete. These symptoms may steal on gradually, or suddenly make their appearance. In irritable persons the suffering is considerable—in those of an opposite habit, they are comparatively slight. When retention occurs, its usual consequences follow—distention of the bladder to a certain point, pain above the pubes, dribbling of urine, and so on.

A slight degree of frequency of making water is common enough in gonorrhœa, and need not occasion alarm. But when the urine is frequently passed, in small quantities only, and with spasm in the act of passing it, the surgeon should beware, for inflammation of the neck of the bladder is occurring.

What is to be done? Should the catheter be passed at once, or should antiphlogistic remedies be previously adopted? This is a question which has occasioned much dispute. Yet probably the following practical rule is the safest and the best.

If the retention is not complete, let the patient immediately be cupped in the perineum, and placed in the warm bath. Calomel, with tartar-emetic and opium—and salines with diaphoretics, and especially with Dover's powder, should be given—opiate enemata used—and the bowels cleared out by senna, combined with colchicum. The cupping and the bath should be repeated more or less, in accordance with the circumstances of the case. These means will soon enable us to determine whether the retention will or will not yield to antiphlogistic measures. If it does, so much the better—if it does not, the catheter must be employed.

The same rule may be laid down in cases of complete retention. But the time for experiment is brief in this case. Here, also, general bleeding is sometimes beneficial.

I must say that, in the majority of cases, the catheter is required at last. Then why not, it may be enquired, have recourse to it at once? The reply is satisfactory to my mind. As retention of urine, in this instance, depends on inflammatory action, nothing can be lost, and much may be gained, by the adoption of depleting and tranquillizing measures. Though the instrument is used afterwards, it is used under more favourable circumstances, with a slighter probability of doing mischief than when pushed through parts in a state of extreme irritation and excitement. The judgment of the surgeon is seen in his discrimination of the period beyond which delay would be disadvantageous.

I will merely mention two cases in illustration of the preceding observations.

*Case.* I was requested by my friend Mr. Hering, of Foley Place, to see a gentleman then labouring under retention of urine. He had had acute gonorrhœa, and had taken, as I understood, much exercise. When I saw him, the retention of urine was complete. There was great pain above the pubes—much fulness in the region of the bladder—great spasm in the situation of its neck. The pulse was full—the cheek flushed—the skin hot.

We immediately agreed on bleeding the patient to syncope—put him in the hip-bath, (a general warm-bath could not at the instant be procured,) and gave salines with ipecacuanha and Dover's powder, to maintain the nausea and perspiration that the bleeding had produced. An opiate enema was also given.

In a few hours afterwards I again met Mr. Hering. No water had been passed, the desire to evacuate the bladder was incessant, and the attempts as painful as fruitless. It was evident that the urine must be drawn off. I attempted to introduce the smallest silver catheter.* The spasmodic contraction at the membranous part of the urethra rendered it impossible to enter the bladder without the employment of force. I laid aside the catheter, and took the very finest catgut bougie, and passed it very gently and stealthily on, rotating it on its axis, and taking care to pull firmly on the penis, in order to render the canal of the urethra as straight as extension could make it.* With a little patience the catgut bougie passed into the bladder. After retaining it for a few minutes in the part, I withdrew it, and introduced in a similar way a rather larger wax bougie. This also was retained for a short time, when the patient was directed to endeavour to make water, and then, in the very midst of his efforts, the bougie was suddenly withdrawn. No urine, however, followed. I now took a very small gum catheter, deprived of its stilet, and introduced it like a bougie. It entered the bladder with little difficulty, and the water was drawn off.

I need not pursue the subsequent treatment. Salines with diaphoretics and sedatives—opiate injections—calomel and opium at night, and senna with colchicum in the mornings—leeches to the perineum, and abstraction of blood from the leech-bites by the application of cupping-glasses;—these measures gradually allayed the irritation and inflammation of the neck of the bladder. In about ten days the gentleman began to get about. Discharge remained, but we were very cautious in our interference with it. Ultimately this was checked by small doses of cubebs with nitre and soda, and injections of the acetate of lead.

*Case.* About three weeks ago a gentleman consulted me under the following circumstances. He was an officer in a regiment stationed in India, and was now in England on sick-leave. About a month prior to my seeing him he contracted his first gonorrhœa. He went to an eminent surgeon in London, who immediately ordered him injections of the sulphate of zinc and cubebs. The discharge soon diminished, and in

* The regular sizes of the instrument-makers do not descend low enough in the scale. A very good and convenient silver catheter can be made two sizes below the No. 1 of the shops. The best catheters are undoubtedly those recommended and used by Sir Benjamin Brodie. The catheter is shorter, and describes a smaller portion of a circle at the curve than those commonly employed. It is also set in a wooden handle, which makes it very manageable.

* This is the great rule to be attended to in the introduction of *bougies*. We have no power of directing them, and we must make the urethra as straight as possible, that their point may not hitch in any of its culs-de-sac or dilatations.

about three weeks from the commencement of the complaint disappeared.—But at this time he experienced a desire to pass his water frequently, made only trifling quantities at a time, and had much spasm in the act of micturition in the perineum, as well as pain above the pubes. He mentioned these symptoms to the surgeon who attended him, but was told they were of no consequence. They increased, and when the gentleman applied to me, he could only make about a wine-glassful of water at a time, was obliged to pass that very frequently indeed, had very great spasm at the neck of the bladder, and fulness over the pubes.

I ordered the gentleman to be cupped immediately, and to take the hot-bath—gave him calomel and opium at night, senna, &c. in the morning, and demulcents with sedatives during the day—and prohibited his rising more than was absolutely indispensable from the sofa. The cupping was repeated two or three times, and the other means were vigorously followed up. But after some days no material improvement had ensued, and reluctantly I was compelled to employ the catheter.

A No. 5. could not be passed. After giving a more abrupt curve to a No. 2, such a curve as is required in cases of enlargement of the prostate, I introduced that sized catheter into the bladder—yet not without some difficulty, for the muscles of the membranous part of the urethra contracted very violently. A large quantity of high-coloured urine was drawn off.

On making an examination by the rectum, both lobes of the prostate were felt greatly enlarged, and evidently projecting into the bladder. They were extremely tender upon pressure. The whole membranous part of the urethra was thickened, indurated, and tender when pressed. I was much afraid, from the great size of the prostate and its excessive tenderness, that abscess would occur in it.

The cupping on the perineum was repeated — calomel was given so as slightly to affect the mouth—salines, with hyosciamus, &c. prescribed—purgatives continued—and the hip-bath, with frequent injections of warm water into the rectum, and occasional opiate injections followed up.

It was necessary to introduce the catheter daily for a week. After the bladder had been once emptied even the power of voiding the smallest quantity was lost. At the end of the week, the gentleman gradually regained the ability to pass his water, and now empties the bladder perfectly. The enlargement and induration of the prostate have diminished, but they have not yet (March 2) disappeared. The urine is passed in a great measure by spurts, and no doubt some constriction of the membranous part of the urethra exists. The urethral discharge and ardor urinæ returned as the inflammation at the neck of the bladder was relieved. At this time, too, the absorbents of the penis became inflamed, and an indurated cord on its dorsum led to a circumscribed induration of the prepuce behind the glans. This is almost gone, and the patient is gradually, though by no means rapidly recovering.

The length to which this paper has extended forbids the indulgence in further observations, as well as the introduction of other cases. I would simply mention that, taken in connexion with the cases and remarks already published, these shew, or serve to shew, that the inflammatory consequences of gonorrhœa are sufficiently frequent and important to induce a judicious surgeon to pause before he pursues that indiscriminate, stimulating, and empiric mode of treatment, so constantly employed in this complaint.

My object has been to dwell upon the circumstance—that, although gonorrhœa is a specific inflammation of the urethral mucous membrane, yet, like other specific inflammations, it displays a remarkable tendency to give rise to ordinary inflammation in contiguous or associated tissues. I have also insisted on what is consistent with reasoning, and I believe, with fact, that that tendency is heightened, greatly heightened, by the administration of general or local stimulants during the inflammatory stage of clap.

Far be it from me to recommend decided antiphlogistic treatment for simple gonorrhœa. But whilst there is *much* pain, and if there are *any* symptoms of inflammation of the lacunæ—the cellular tissue of the penis, or of its absorbents—the corpus spongiosum—the perineum—the prostate—the bladder—or the testicle, I would recommend the surgeon to discard all idea of arresting the discharge, and to direct his attention to averting the impending mischief. In our profession, to be successful, we should first be safe.

---

### Advantages of possessing no Advantages.

Our Dublin contemporary has latterly talked much in King Cambyses' vein. Every number now breathes battle, and murder, and sudden death, to the misguided wretches, who are disposed to question the purity and excellence of the Dublin Colleges. We are really alarmed when we read such manifestoes as this :—

"We are fully aware of the designs of our enemies, and prepared to meet them; we are girded for the fight, and that fight shall not be with shadows, but hilt to hilt, in the face of our fellow citizens."

Ave Maria! Why Ancient Pistol was nothing to these gentlemen, who no doubt will not only kill their enemies but eat them. How valiant is the declaration, that they don't intend to fight with shadows. No adversaries short of men in real buckram will satisfy such fiery spirits. The White-boys and the Terry Alts had best come over the water at once—their occupation's gone.

It would be curious to ascertain the causes of this infusion of ferocity in our erst peaceable contemporary. Hitherto displaying the philosophical placidity of science, he on a sudden gnashes his teeth and bites his tongue, as though he were in a fit of the falling sickness. To use an ocular pun, we should suppose a new humour had been found in him—an aqua Jacobi. Yet no aqueous humour, but ardent and inflammable as the purest potsheen of Tipperary.

We have no intention of entering the lists with our contemporary. He is far too bloody-minded for us. We therefore leave him to fight his fights, and annihilate his already annihilated enemies. This sort of prowess has been commemorated :

> "Moved at the sight, the King grew vain,
> And thrice he routed all his foes, and
> thrice he slew the slain."

But we are anxious to direct attention to an important discovery of our contemporary's. Some persons have supposed that the metropolis possesses many advantages for literary undertakings. Nay, so prevalent is this absurd idea, that men actually repair to it for the purpose of conducting them with more effect. It has been supposed too by reflecting people, that professional experience and attainments are usually best obtained and displayed in the vast theatre for observation and for enterprise that a great metropolis presents. The poets have been the folks who have babbled of green fields, but even the poets have generally found that, however sweet those fields might be, the richest soil and the brightest landscape were in Paternoster Row. The wits have ever congregated in the coffee-house, and not under the hedge. These, however, were the wits of days gone by—scurvy fellows, like Johnson, Dryden, Pope, Addison, or Goldsmith. The modern wits of Dublin are of another way of thinking. Speaking of a medical journal, they sagaciously observe :—

"Another circumstance is favorable to its success. The editors do not live in London. They are removed from the temptations to which they should be daily exposed in that great market, where reputation, good, bad, or indifferent, is bought, or sold, or exchanged, as openly as their fish at Billingsgate, or their cabbages in Covent Garden: we hail its appearance as evidence that the spell is broken, the 'tall bully may lift his head, and lie,' but the day of 'cushioning' is gone. Edinburgh, Dublin, Glasgow, Newcastle, York, Leeds, Manchester, Liverpool, Birmingham, and Bristol, are Londons now."

These towns may be "Londons" in the estimation of their own inhabitants, for the Lilliputians do not think meanly of Lilliput; but they are not Londons notwithstanding. We will not argue this foolish question with our petulant contemporary. But we would ask him —why stop at Dublin, and York, and Manchester? If reputations are bought and sold like cabbages in London, why should they not be bought and sold like potatoes in his own town? Are parties so pure in the Green Isle? Are Irishmen now so united, that no favouritism, no jealousy can be developed in that land of calm enquiry? Philosophical people! no bickerings, no religious animosities exist amongst you. Occupied solely in the dignified abstractions of science, you resemble the clear, pure, mild, and mellow spirit that you quaff; and, animated by whiskey and universal philanthropy, you flourish your shillelahs and break each other's heads, without those sordid motives of provocation or resentment that disgrace the calculating denizens of London.

That such is the tranquil condition of the medical profession in Dublin must be evident, from the following statements of our contemporary. The intelligent reader will perceive at once how admirably adapted such a state must be, for the support of candid and independent criticism.

"Once for all, we declare most solemnly, and without the slightest apprehension of refutation, that the efforts of the real friends of reform and improvement in Dublin, have had but one difficulty to encounter in the course of many years' labour in the cause, and that difficulty has been the intrigues, vexatious interruptions, and dishonest practices of the mock reformers. By their various subterfuges, a most formidable barrier has been raised against real reform, and a most convenient veil drawn over the delinquencies of the very men who intend to impute to others the shameful practices of which they stand convicted themselves. Among some of these gentlemen, we recognize certain heartless men, who coolly and systematically mix themselves up with controversies respecting medical education and similar matters, without feeling the slightest interest in them, or caring in the slightest degree for the result. Their end is to keep their names before the public, and to postpone that oblivion into which they feel themselves naturally sinking."

Placid as such society is, and calculated for the dispersion of every thing like partiality, we think our contemporary might have pushed his idea a little farther. If London editors are bad, because party and traffic thrive in it, surely the more remote the spot from the contaminating influences of either, the brighter and purer the editor must be. According to his own shewing, Dublin, and Liverpool, and Manchester are Londons now. But, alas, if they inherit the greatness, they will also inherit at least a portion of the vices of their prototype. Better, then, to place the nursery in the Hebrides, or the Isle of Man, or somewhere far distant from the seats of human thoughts and human feelings.*

---

* Men would do well to think before they write. What are the Dublin Editors? Do they not live in the METROPOLIS of

> "The first flower of the Earth—the first gem of the Sea?"

Have we ever accused them of *venality* on that account? Have we ever used a single disrespectful word towards them? Yet here is our reward! The *onus probandi* lies with the accuser. We cannot disprove that which does not exist; but we call upon our Dublin contemporary to adduce the shadow, either of proof or *probability*, that the conductors of this Journal ever condescended to bribery, corruption, or any species of venality. The accusation in question, would, if unsupported by proof, subject a man, in private life, to disgrace and contempt. As an assassin of character, we dare him to the proof of an iota of the grave charge which he has preferred. Conscious of our integrity, we put our accuser on his trial—substantiation of the crime in us—or conviction of unfounded slander in himself.

